# 5-azacytidine inhibits nonsense-mediated decay in a MYC-dependent fashion

**DOI:** 10.15252/emmm.201404461

**Published:** 2014-10-15

**Authors:** Madhuri Bhuvanagiri, Joe Lewis, Kerstin Putzker, Jonas P Becker, Stefan Leicht, Jeroen Krijgsveld, Richa Batra, Brad Turnwald, Bogdan Jovanovic, Christian Hauer, Jana Sieber, Matthias W Hentze, Andreas E Kulozik

**Affiliations:** 1 Molecular Medicine Partnership Unit, European Molecular Biology Laboratory, University of HeidelbergHeidelberg, Germany; 2 Department of Pediatric Oncology, Hematology and Immunology, University of HeidelbergHeidelberg, Germany; 3 European Molecular Biology LaboratoryHeidelberg, Germany; 4 Department of Mathematics and Computer Science, University of Southern DenmarkOdense, Denmark; 5 Centre for Molecular Biology of the University of Heidelberg, University of HeidelbergHeidelberg, Germany

**Keywords:** 5-azacytidine, MYC, nonsense-mediated decay, premature termination codons

## Abstract

Nonsense-mediated RNA decay (NMD) is an RNA-based quality control mechanism that eliminates transcripts bearing premature translation termination codons (PTC). Approximately, one-third of all inherited disorders and some forms of cancer are caused by nonsense or frame shift mutations that introduce PTCs, and NMD can modulate the clinical phenotype of these diseases. 5-azacytidine is an analogue of the naturally occurring pyrimidine nucleoside cytidine, which is approved for the treatment of myelodysplastic syndrome and myeloid leukemia. Here, we reveal that 5-azacytidine inhibits NMD in a dose-dependent fashion specifically upregulating the expression of both PTC-containing mutant and cellular NMD targets. Moreover, this activity of 5-azacytidine depends on the induction of MYC expression, thus providing a link between the effect of this drug and one of the key cellular pathways that are known to affect NMD activity. Furthermore, the effective concentration of 5-azacytidine in cells corresponds to drug levels used in patients, qualifying 5-azacytidine as a candidate drug that could potentially be repurposed for the treatment of Mendelian and acquired genetic diseases that are caused by PTC mutations.

See also: **A Shao & MF Wilkinson** (December 2014)

## Introduction

Nonsense-mediated mRNA decay (NMD) specifically recognizes and degrades transcripts with premature termination codons (PTCs) in a translation and splicing-dependent manner (Hentze & Kulozik, 1999; Maquat, 2004). PTCs may be introduced into mRNAs by mutations, transcriptional errors, or aberrant splicing (Holbrook *et al*, 2004). Recognition of PTC-containing mRNAs and their targeting for degradation requires a set of conserved NMD effectors, which include the Up-frame shift (UPF) proteins UPF1, UPF2 and UPF3B; exon junction complex (EJC) proteins Y14, MAGOH, EIF4AIII and BTZ (MNL51); and the SMG1-SMG9 proteins (Yamashita *et al*, 2001; Kashima *et al*, 2006). When the ribosome reaches a PTC, interaction of the release factors eRF1 and eRF3 with downstream EJCs bridged by the UPF proteins triggers the phosphorylation of UPF1 and subsequent degradation of the mRNA [reviewed in (Holbrook *et al*, 2004; Chang *et al*, 2007; Bhuvanagiri *et al*, 2010)]. Although the general pathway of NMD is conserved among different species, important differences have been identified. Studies in mammals have suggested alternative branches of the NMD pathway, which differ in the dependence on the cofactors UPF2, EJC core components and UPF3B, but converge at the point of UPF1 phosphorylation (Gehring *et al*, 2005).

The importance of NMD is also reflected in its ability to modulate expression of many physiological transcripts, referred to as “endogenous NMD targets”, involved in various cellular processes, such as haematopoietic cell differentiation or the maintenance of chromosome structure and function (Mendell *et al*, 2004; Isken & Maquat, 2008). Inhibition of NMD stabilizes mRNAs allowing cells to effectively respond to stress (Karam *et al*, 2013). Furthermore, approximately one-third of human Mendelian diseases are estimated to be caused by PTCs (Holbrook *et al*, 2004). The degradation of PTC-mutated mRNAs limits the synthesis of their encoded C-terminally truncated proteins. When such truncated proteins act in a dominant negative fashion, NMD can protect from severe disease in heterozygous carriers, as is exemplified in β-thalassemia (Holbrook *et al*, 2004). By contrast, when the truncated proteins are (partially) functional, as is the case with some PTC mutations causing cystic fibrosis (CF), Duchenne muscular dystrophy (DMD) and some types of cancer, NMD can aggravate the disease phenotype (Holbrook *et al*, 2004, 2006; Karam *et al*, 2008; Bhuvanagiri *et al*, 2010). Because of its importance in modulating the phenotype of PTC-related Mendelian and somatic diseases such as cancer, NMD represents an attractive target for the development of novel treatment strategies. Some small molecules have previously been used to study the mechanism of NMD in experimental systems. These include (i) compounds that induce translational readthrough such as the aminoglycosides G418 and gentamycin (Keeling & Bedwell, 2002, 2011; Dranchak *et al*, 2011), (ii) general translation inhibitors such as cycloheximide and anisomycin (Belgrader *et al*, 1993; Carter *et al*, 1995; Li *et al*, 1997) and (iii) direct inhibitors of NMD proteins such as wortmannin and pateamine A (Usuki *et al*, 2004; Dang *et al*, 2009). Readthrough agents induce the insertion of a near-cognate transfer RNA at the position of a stop codon, which results in the incorporation of an amino acid in place of the stop codon into the nascent peptide and could potentially be used to treat PTC-mediated diseases (Burke & Mogg, 1985; Linde *et al*, 2007b; Linde & Kerem, 2008; Keeling *et al*, 2012). However, the readthrough agent gentamycin, which is clinically used as an antibiotic, cannot be used for this purpose, because it is too toxic at concentrations that modulate NMD efficiency (Mingeot-Leclercq & Tulkens, 1999; Guthrie, 2008). The indole derivative PTC124 has been developed as a readthrough-promoting agent for PTC mutations (Welch *et al*, 2007), and it is currently in late-stage clinical trials for the treatment of DMD and CF (Finkel *et al*, 2013; Kerem *et al*, 2014). Amlexanox is a small molecule with both NMD inhibitory and readthrough-promoting activity. However, the exact mechanism of action of amlexanox is still unknown (Gonzalez-Hilarion *et al*, 2012). The combination of the readthrough-promoting compound G418 and the NMD modulator NMDI14 was shown to stabilize mRNAs with PTCs and restore expression of full-length p53 protein (Martin *et al*, 2014). These studies indicate that NMD inhibition can principally be achieved pharmacologically, motivating the search for new compounds with clinically appropriate effectivity/safety profiles. We thus screened for drugs that inhibit NMD and are that are already clinically approved for other purposes. This work identifies 5-azacytidine as a dose-dependent novel NMD inhibitor and characterizes its mechanism of action, which depends on MYC activation.

## Results

### 5-azacytidine specifically inhibits NMD

**Figure 1:**
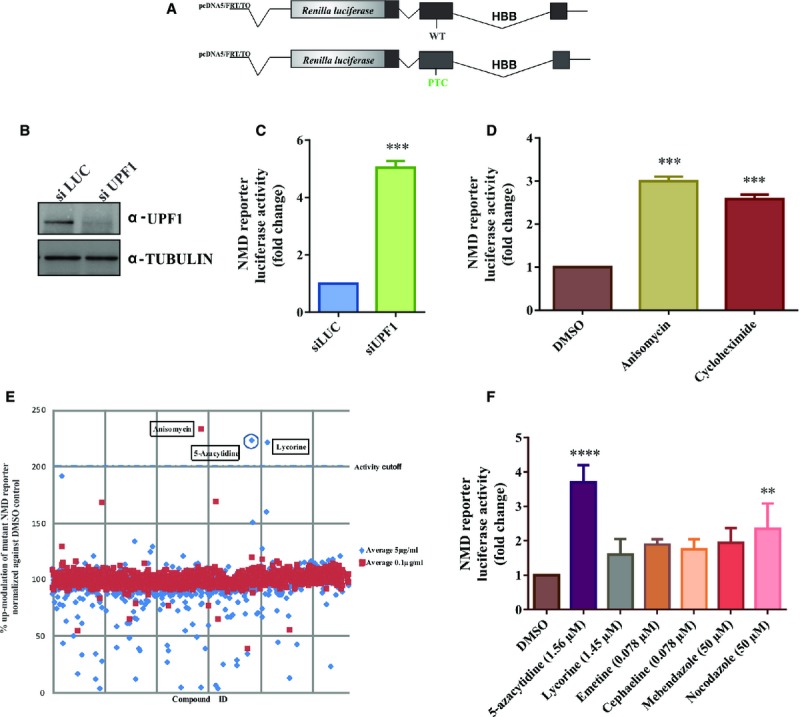
5-azacytidine inhibits NMD Schematic representation of the NMD reporter, which contains a either a wild-type or a PTC-mutated HBB gene fused to a renilla luciferase gene and which was used to stably transfect HeLa cells.Western blot documenting the efficient depletion of UPF1 protein in HeLa cells stably expressing the reporter construct.Upregulation of reporter luciferase activity following siRNA-mediated depletion of UPF1. Student's *t*-test was performed to analyze the significance, *N* = 3 and ***P = 0.0006. Each bar represents average ± SD.Upregulation of reporter luciferase activity following treatment with known NMD inhibitors, which identified anisomycin and cycloheximide as suitable positive controls for the high-throughput screen. One-way ANOVA followed by Holm–Sidak multiple comparisons test was performed to analyze the significance, *N* = 3 and ***P = 0.0003 for anisomycin and ***P = 0.0006 for cycloheximide. Each bar represents average ± SD.Graphical representation of the primary screening data. HeLa cells stably expressing the reporter were treated with 5 μg/ml (blue diamond) and 0.1 μg/ml (red square) concentrations of compounds contained in the Prestwick library along with DMSO as a negative control and anisomycin as a positive control. Following 18 h of treatment, cells were lysed and the renilla luciferase luminescence signal was detected. Data represent the average of two biological replicates. Compounds that resulted in > 200% reporter activity are indicated.Assessment of reporter activity at concentrations that have been identified to be optimal by dose–response titrations (see Supplementary Fig S2) of hits from the high-throughput screen [5-azacytidine (1.56 μM); lycorine (1.45 μM); emetine (0.078 μM); cephaeline (0.078 μM); mebendazole (50 μM); nocodazole (50 μM)]. One-way ANOVA followed by Holm–Sidak multiple comparisons test was performed to analyze the significance, *N* = 3 and ****P < 0.0001 for 5-azacytidine and **P = 0.0080 for nocodazole. Each bar represents average ± SD. Source data are available online for this figure.

We first developed and validated a chemiluminescence-based high-throughput screening assay in HeLa cells stably expressing an NMD reporter that has previously been generated in our laboratory. This reporter consists of a hemoglobin beta subunit (HBB) gene either with a PTC at position 39 of the open reading frame (ORF) in exon 2 or with the normal sequence, fused to a renilla luciferase gene (Fig1A). When NMD is active, the mRNA of the PTC-mutated fusion transcript is degraded and the renilla luciferase activity is low (Boelz *et al*, 2006). Inhibition of NMD results in the upregulation of nonsense-mutated mRNA levels and increased luciferase activity of the reporter. We validated this reporter system by monitoring the effect of siRNA-mediated depletion of the essential NMD factor UPF1. Downregulation of UPF1 protein resulted in the expected upregulation of the PTC-mutated reporter (Fig1B and C). We further validated the reporter by testing known inhibitors of NMD. Among the various controls tested, anisomycin and cycloheximide showed a more than twofold upregulation of the luciferase-based NMD reporter at low concentrations that only partially inhibit general translation (Fig1D and Supplementary Fig S1).

We then used the reporter system to perform a screen of the Prestwick library (Prestwick Chemical), containing 1,120 clinically licensed drugs or compounds in advanced clinical development. HeLa cells stably expressing the NMD reporter were seeded in 384 well plates and treated either with the controls (positive: anisomycin; negative: DMSO) or with the compounds from Prestwick library at concentrations of 0.1 or 5 μg/ml for 18 h (Fig1E). The majority of the compounds screened showed little or no effect on the NMD reporter at either concentration. By contrast, 5-azacytidine (5 μg/ml) and lycorine (5 μg/ml) yielded a more than 200% up-modulation of luciferase activity, and treatment with emetine, cephaeline, mebendazole and nocodazole resulted in a 150–200% up-modulation of the NMD reporter (Fig1E). All six compounds that showed an effect in the primary screen were then selected for secondary screening using HeLa cells stably expressing either the wild-type or the PTC-mutated HBB reporter (Supplementary Fig S2). In this secondary screen, dose–response curves were generated using an 11-point twofold serial dilution starting at 100 μM. Further, viability of the cells was measured in parallel by monitoring the intracellular ATP concentrations. These analyses demonstrated that 5-azacytidine is effective at concentrations starting from 0.5 μM up to a maximum of around 10 μM. Above this concentration, there was a reduction in luciferase activity resulting in a bell-shaped dose–response curve. This is most likely due to toxicity associated with the drug at higher concentrations. Further, this analysis revealed that 5-azacytidine specifically acts on NMD and does not increase the expression of the wild-type reporter construct, also excluding the possibility of unspecific effect on luciferase enzyme activity. The other hits of the primary screen show marginal effects at lower concentrations (lycorine, nocodazole) or effective only at higher concentrations (50 μM) (cephaeline, emetine, mebendazole), which are not practical for clinical use (Supplementary Fig S2). Retesting these compounds at the concentration at which the maximum signal for the PTC mutant had been detected in the dose–response titration confirmed the results of the primary screen and revealed a statistically significant increase of the expression of the PTC-mutated transcript following treatment with 5-azacytidine and nocodazole, while there was a non-significant trend for the other compounds tested (Fig1F). Additionally, it is interesting to note that 5-azacytidine results in the synthesis of approximately 40% of the wild-type protein at a concentration of 1.56 μM (Supplementary Fig S3A). 5-azacytidine thus emerges as a promising inhibitor of NMD identified from the Prestwick library.

### Chemical specificity of 5-azacytidine

**Figure 2:**
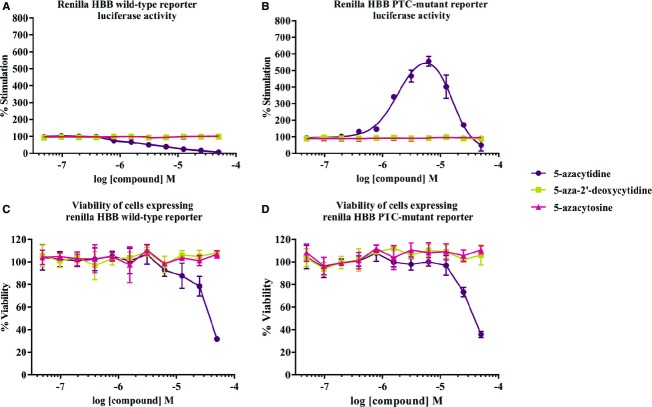
Chemical specificity of 5-azacytidine as an NMD inhibitor HeLa cells stably expressing HBB wild-type and PTC-mutant renilla luciferase reporters were used to study the dose–response effect of nucleoside and nucleotide analogues (see Table1).A, BAssessment of the stimulation of luciferase activity of the HBB wild-type or PTC-mutant reporters by treatment with 5-azacytidine, 5-azacytosine or 5-aza-2′-deoxycytidine. The *x*-axis represents the serial dilution of the compounds, and the *y*-axis shows percentage (%) stimulation of luciferase activity.C, DViability of HeLa cells expressing the wild-type or mutant HBB reporters was measured after 18 h of treatment with serial dilutions of 5-azacytidine, 5-azacytosine or 5-aza-2′-deoxycytidine.

We next analyzed whether any chemically related analogues of 5-azacytidine could also inhibit NMD. We used stable cell lines expressing wild-type or PTC-mutated reporter constructs and treated them with serial dilutions of 20 chemically related nucleoside/nucleotide analogues (Table1), some of which are in clinical use for the treatment of cancer and viral diseases, and performed luciferase and cytotoxicity assays (Fig2A–D). Our analyses showed that none of the drugs, even the chemically closely related 5-aza-2′-deoxycytidine and 5-azacytosine, display any effect on wild-type or NMD reporter, unlike 5-azacytidine which specifically stimulates the luciferase activity encoded by the PTC-mutated reporter by more than 200% already at a concentration of 1 μM (Fig2A and B). These results document the high degree of chemical specificity of 5-azacytidine in inhibiting NMD. Hence, for our further analyses, we have chosen a concentration of 1.56 μM of 5-azacytidine at which we observed minimal cytotoxicity and robust up-modulation of NMD reporter activity (Fig2C and D).

**Table 1:** Summary of the results of dose titration experiments with 20 nucleoside/nucleotide analogues on the expression of the PTC-mutated and the wild-type reporter constructs

Compound	Upregulation of PTC-mutant reporter luciferase activity	NS39 (EC μM)	Upregulation of wild-type reporter luciferase activity	Wild-type (EC μM)
Cytidine	NO	> 50	NO	> 50
2′-deoxycytidine	NO	> 50	NO	> 50
3′-deoxycytidine	NO	> 50	NO	> 50
5-azacytosine	NO	> 50	NO	> 50
5-azacytidine	YES	1.56	NO	> 50
5-aza-2′-deoxycytidine	NO	> 50	NO	> 50
2′,3′-dideoxycytidine	NO	> 50	NO	> 50
2′-deoxycytidine	NO	> 50	NO	> 50
2′-O-methylcytidine	NO	> 50	NO	> 50
3′-deoxycytidine	NO	> 50	NO	> 50
3′-O-methylcytidine	NO	> 50	NO	> 50
6-azathymine	NO	> 50	NO	> 50
6-azauracil	NO	> 50	NO	> 50
Cytarabine	NO	> 50	NO	> 50
Gemcitabine	NO	> 50	NO	> 50
l-cytidine	NO	> 50	NO	> 50
N4-aminocytidine	NO	> 50	NO	> 50
Thymidine	NO	> 50	NO	> 50
Uridine	NO	> 50	NO	> 50
Zebularine	NO	> 50	NO	> 50

EC, effective concentration.

### 5-azacytidine increases the abundance of nonsense-mutated mRNAs at the post-transcriptional level

**Figure 3:**
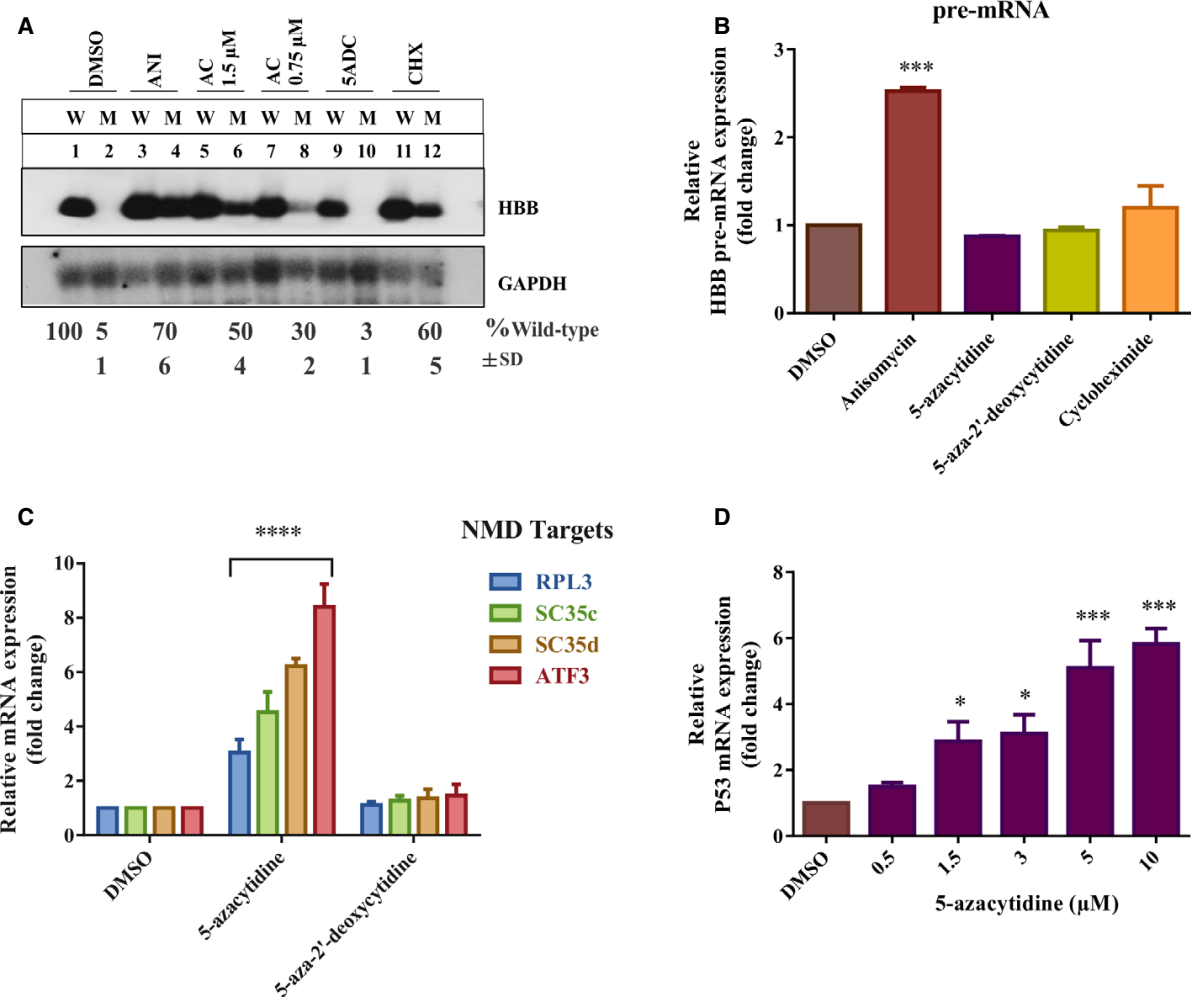
5-azacytidine acts post-transcriptionally Northern blot of total cellular RNA of HeLa cells stably expressing wild-type (W) or PTC-mutated (M) HBB genes, following the treatment with DMSO, 5-azacytidine (AC) at concentrations of 0.5 and 1.56 μM, anisomycin (ANI), 5-aza-2′-deoxycytidine (5ADC) or cycloheximide (CHX) for 18 h. GAPDH mRNA was assayed as a loading control and used for normalization. The expression of PTC-mutated HBB reporter mRNA is shown in % of wild-type with the standard deviation (SD) of at least three independent experiments.qRT-PCR analysis of reporter pre-mRNA of the same RNAs as shown in panel (A). GAPDH pre-mRNA is used for normalization. One-way ANOVA followed by Holm–Sidak multiple comparisons test was performed to analyze the significance, *N* = 3 and ***P = 0.0002 for anisomycin. Each bar represents average ± SD.qRT-PCR analysis of the endogenous NMD targets RPL3, SC35c, SC35d and ATF3 following the treatment of HeLa cells for 18 h with DMSO, 5-azacytidine or 5-aza-2′-deoxycytidine. The fold change on the *y*-axis represents the relative quantification of transcripts versus GAPDH mRNA, which is used as a normalization control. The signal detected in DMSO-treated cells is set as 1. Two-way ANOVA followed by Newman–Keuls multiple comparison test was performed to analyze the significance, *N* = 3 and ****P < 0.0001 for RPL3, SC35c, SC35d and ATF3 with 5-azacytidine treatment. Each bar represents average ± SD.qRT-PCR analysis of Calu-6 cells (carrying a homozygous PTC mutation of the P53 gene) following treatment with either DMSO or increasing concentrations of 5-azacytidine for 18 h. The fold change on the *y*-axis represents the relative quantification of PTC-mutated P53 transcript versus GAPDH mRNA, which is used as a normalization control. The signal detected in DMSO-treated cells is set as 1. One-way ANOVA followed by Holm–Sidak multiple comparisons test was performed to analyze the significance, *N* = 3 and *P = 0.03 and 0.02 for 5-azacytidine (1.5 and 3 μM) and ***P = 0.0008 and 0.0003 for 5-azacytidine (5 and 10 μM) respectively. Each bar represents average ± SD.

To characterize the mechanism of action of 5-azacytidine, we examined its effect on the abundance of PTC-mutated mRNA. Stable HeLa cell lines expressing wild-type or PTC-mutated HBB reporters were incubated for 18 h either with 5-azacytidine, anisomycin or cycloheximide as positive controls, or DMSO and 5-aza-2′-deoxycytidine as negative controls. Total RNA was analyzed by Northern blotting (Fig3A). Treatment with 5-azacytidine increased the steady state level of the NMD reporter sixfold at a concentration of 0.75 μM (Fig3A, compare lanes 1 & 2 with 7 & 8), and tenfold at 1.5 μM (compare lanes 1 & 2 with 5 & 6). In comparison, the positive controls anisomycin and cycloheximide yielded a 14-fold and 12-fold stimulation, respectively (lanes 3 & 4 and 11 & 12). By contrast, the negative control 5-aza-2′-deoxycytidine did not stimulate the expression of the reporter mRNA when compared to the solvent DMSO (compare lanes 1 & 2 with 9 & 10). Furthermore, as 5-azacytidine is known to remodel chromatin and might thus influence transcription (Christman, 2002), we tested whether the effect of 5-azacytidine is post-transcriptional and thus consistent with the postulated effect on NMD. We quantified the HBB pre-mRNA via qRT-PCR, using GAPDH pre-mRNA as a normalization control. This analysis showed that 5-azacytidine did not affect pre-mRNA levels, hence confirming the effect of 5-azacytidine to be post-transcriptional. By contrast, anisomycin, which is known to affect both, transcriptional and post-transcriptional steps of gene expression (Ronkina *et al*, 2011), increased the pre-mRNA levels of the reporter by approximately 2.5-fold (Fig3B).

Considering that 5-azacytidine inhibits NMD of a chimeric PTC reporter gene construct, we next tested whether it also modulates the expression of the so-called endogenous NMD targets (Mendell *et al*, 2004). We analyzed the responses of several of such NMD targets by qRT-PCR analysis of total cellular mRNA of 5-azacytidine-treated cells. All of these targets responded to 5-azacytidine treatment by approximately threefold to eightfold upregulation, whereas the negative control 5-aza-2′-deoxycytidine did not alter expression relative to DMSO (Fig3C). The NMD-insensitive isoforms of the tested endogenous NMD targets showed no upregulation upon 5-azacytidine treatment further confirming the specific effect of 5-azacytidine in inhibiting NMD (Supplementary Fig S3B).

To extend these analyses to other mutant NMD targets in a different genetic background, we tested whether 5-azacytidine can specifically upregulate expression of p53 mRNAs carrying a homozygous CGA→TGA PTC mutation at codon 196 in Calu-6 cells (Lehman *et al*, 1991). The 5-azacytidine dose–response curve showed a dose-dependent upregulation of PTC-mutated p53 transcript (Fig3D), further demonstrating that 5-azacytidine specifically modulates the expression of mRNAs that are subject to degradation by NMD.

### 5-azacytidine inhibits NMD neither via readthrough, translation inhibition nor direct inhibition of NMD factors

**Figure 4:**
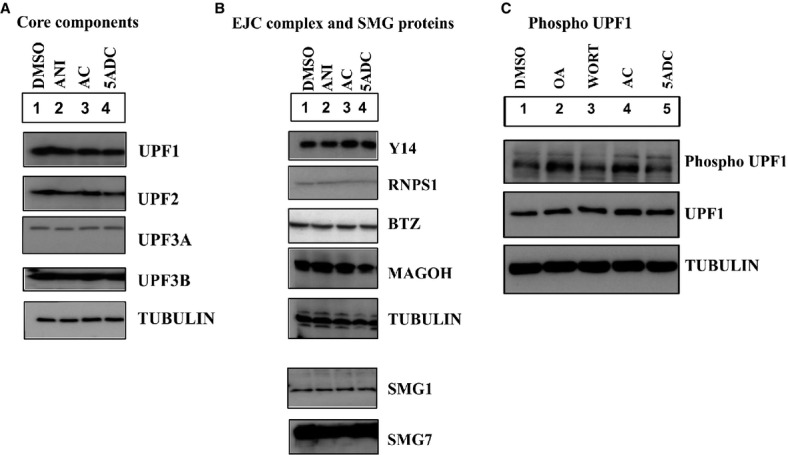
Core NMD factors and EJC complex proteins are unaffected by treatment with 5-azacytidine A–CWestern blot of HeLa cells following treatment with either DMSO as a negative control, anisomycin (ANI) as a positive control or 5-azacytidine (AC) or 5-aza-2′-deoxycytidine (5ADC) for 18 h and staining with antibodies that specifically detect the NMD core components UPF1, UPF2, UPF3A, UPF3B (A); the EJC proteins Y14, MAGOH, RNPS1 and BTZ; and the proteins SMG1 and SMG7 (B), and total and phosphorylated UPF1 following treatment with DMSO, inhibition of UPF1 dephosphorylation with okadaic acid (OA) and inhibition of UPF1 phosphorylation with wortmannin (WORT) (C). Tubulin expression was monitored as a loading control. Source data are available online for this figure.

To define the mechanism by which 5-azacytidine controls NMD, we next tested whether 5-azacytidine might affect NMD protein levels directly. We thus analyzed factors that are known to be necessary for NMD in cells treated with 5-azacytidine, anisomycin or the negative controls (DMSO, 5-aza-2′-deoxycytidine) (Fig4A and B). This analysis showed that the abundance of the NMD core components UPF1, UPF2, UPF3A and UPF3B; the EJC proteins Y14, MAGOH, RNPS1 and BTZ; and SMG1 and SMG7 was unaffected by 5-azacytidine treatment. Furthermore, phosphorylation of UPF1, a key event in triggering NMD, was unaffected by 5-azacytidine (Fig4C).

**Figure 5:**
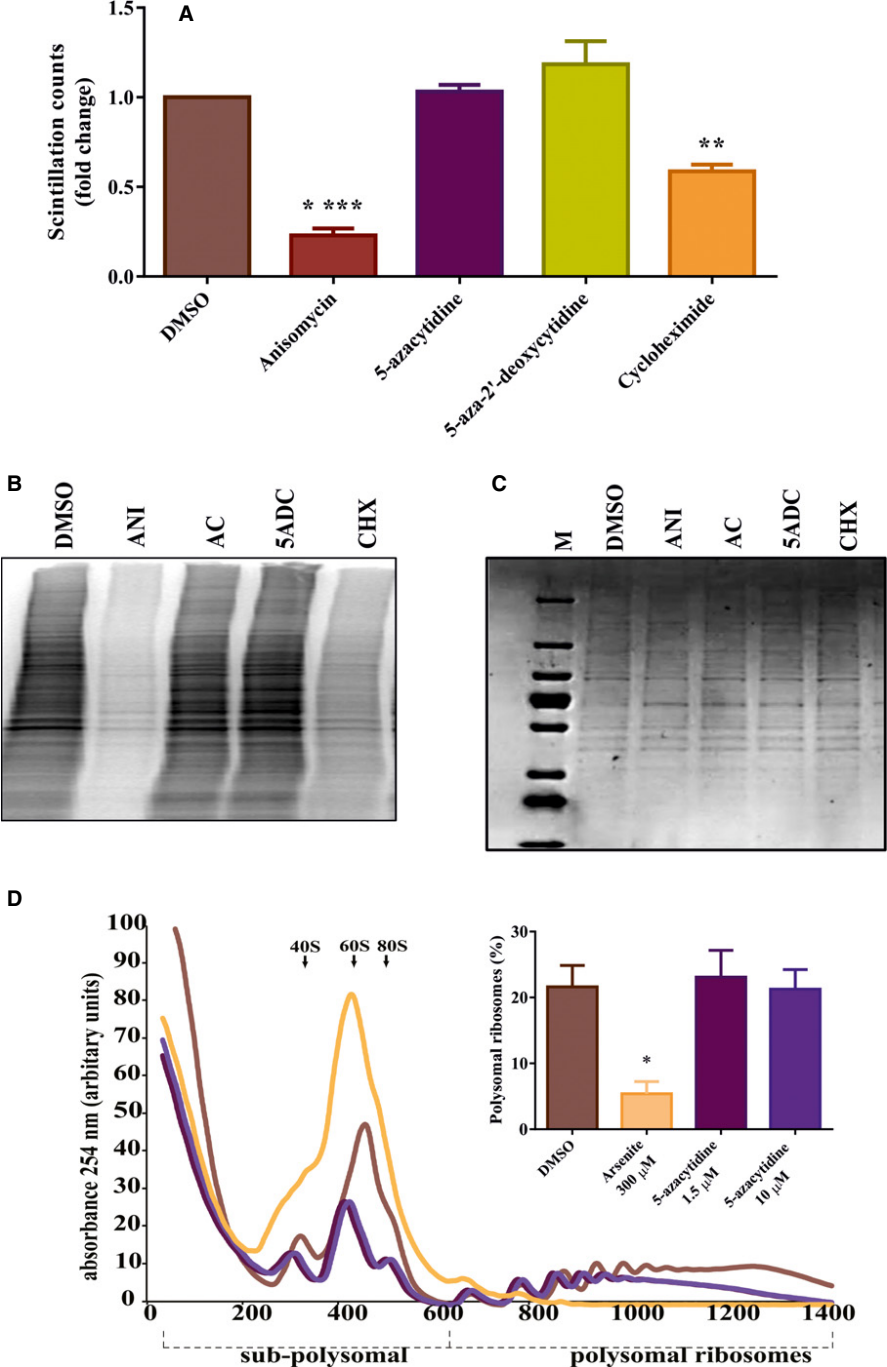
5-azacytidine does not affect *de novo* protein synthesis at concentrations that inhibit NMD Analysis of ^35^S-Met incorporation in HeLa cells following treatment with DMSO, 5-azacytidine, anisomycin, 5-aza-2′-deoxycytidine or cycloheximide. HeLa cells were incubated with the compounds for 18 h, and a pulse of ^35^S-methionine was given for 2 h. ^35^S-Met incorporation was assayed by scintillation counting. One-way ANOVA followed by Holm–Sidak multiple comparisons test was performed to analyze the significance, *N* = 3 and ****P < 0.0001 for anisomycin and **P = 0.0015 for cycloheximide. Each bar represents average ± SD.Autoradiography of a polyacrylamide gel containing the lysates of the cells used for the analysis shown in panel (A) (ANI = anisomycin; AC = 5-azacytidine; 5ADC = 5-aza-2′-deoxycytidine, CHX = cycloheximide).Coomassie staining of the gel shown in panel (B) to control for equal loading.Polysomal profiles were recorded from HeLa cells treated either with the negative control DMSO or with the positive control arsenite, or 1.56 and 10 μM of 5-azacytidine for 18 h. To determine the percentage of polysomal ribosomes, the area below the polysomal part of the curve was divided by the area below the subpolysomal and polysomal parts of the curve and represented as average ± SD in the bar graph. One-way ANOVA followed by Holm–Sidak multiple comparisons test was performed to analyze the significance, *N* = 3 and *P = 0.03 for arsenite treatment. Source data are available online for this figure.

Since 5-azacytidine can cause translational inhibition at high concentrations (Reichman & Penman, 1973) and translation inhibition can down-modulate NMD activity (Carter *et al*, 1995), we tested whether 5-azacytidine interferes with global translation at the concentration (1.56 μM) at which it exerts its maximal inhibitory effect on NMD without affecting cell viability (see Fig2B and D). We thus measured the incorporation of ^35^S-methionine into newly synthesized proteins following treatment with 5-azacytidine, positive controls anisomycin and cycloheximide, or DMSO and 5-aza-2′-deoxycytidine as negative controls. Scintillation counting of trichloroacetic acid (TCA) precipitates showed the expected reduction of ^35^S-methionine incorporation in anisomycin- and cycloheximide-treated cells, whereas *de novo* protein biosynthesis and ^35^S-methionine incorporation were unaffected by 5-azacytidine when compared to DMSO and 5-aza-2′-deoxycytidine (Fig5A). Similarly, autoradiography of proteins following SDS polyacrylamide gel electrophoresis (PAGE) showed a reduced ^35^S-methionine incorporation in anisomycin- and cycloheximide-treated cells, but no negative effect in cells treated with 5-azacytidine, DMSO, or 5-aza-2′-deoxycytidine (Fig5B) when equal loading was ascertained by staining the SDS–PAGE gel with Coomassie blue (Fig5C). Additionally, we performed polyribosomal profile analyses with two different doses of 5-azacytidine. Our results show an almost complete loss of polyribosomes following treatment with arsenite, which was used as positive control. By contrast, when compared to DMSO, which was used as negative control, treatment with 1.56 and 10 μM 5-azacytidine did not change the abundance of polyribosomes (Fig5D). Therefore, the inhibitory effect of 5-azacytidine on NMD cannot be ascribed to an inhibition of translation.

**Figure 6:**
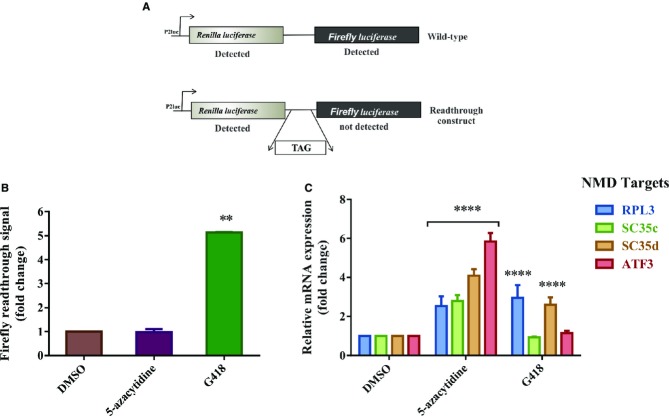
5-azacytidine does not inhibit NMD via a readthrough mechanism Schematic representation of the readthrough reporters. The open reading frames of the renilla and firefly luciferase genes are separated by a spacer that either contains or does not contain a TAG stop codon. In case of the absence of the stop codon both, renilla and firefly luciferase luminescence are detected, while in the presence of the stop codon, the firefly luciferase signal is detected only when there is sufficient translational readthrough.Assay of translational readthrough of HeLa cells that were transiently transfected either with the reporter with or without the stop codon. 24 h following transfection, the cells were treated for 18 h with DMSO (negative control), 5-azacytidine or G418 (600 μM; positive control). After 18 h, cells were harvested and chemiluminescence was measured. The data are shown as fold change of the ratio of firefly/renilla luciferase signal of the construct with the stop codon after normalization of the renilla and firefly luciferase signals generated by the reporter construct without the stop codon. Student's *t*-test was performed to analyze significance, *N* = 3 and **P = 0.0017 for G418. Each bar represents average ± SD.qRT-PCR analysis of the endogenous NMD targets RPL3, SC35c, SC35d and ATF3 in the same cells that were assayed for translational readthrough in panel (B). The fold change on the *y*-axis represents the relative quantification of transcripts following normalization against GAPDH mRNA. The signal detected in DMSO-treated cells is set as 1. Two-way ANOVA followed by Newman–Keuls multiple comparison test was performed to analyze the significance, *N* = 3 and ****P < 0.0001 for RPL3, SC35c, SC35d and ATF3 with 5-azacytidine treatment and ****P < 0.0001 for RPL3 and SC35d with G418 treatment. Each bar represents average ± SD.

We next tested whether 5-azacytidine triggers translational readthrough, the only remaining possibility of the known inhibitory mechanisms of NMD. We used a reporter system consisting of a fusion transcript between the renilla and firefly luciferase open reading frames. In the control version of the construct, the renilla and firefly luciferase ORFs are continuous and uninterrupted by a stop codon; consequently, both renilla and firefly luciferase activities can be measured. In the construct that quantifies translational readthrough, the renilla and firefly luciferase ORFs are separated by a stop codon, and firefly luciferase activity is only detected when readthrough occurs (Fig6A) (Ivanov *et al*, 2008). HeLa cells were transiently transfected with these constructs and treated with either 5-azacytidine, DMSO as a negative control or G418 as a positive control for translational readthrough (Dranchak *et al*, 2011). The “fold change” of readthrough was calculated after normalization against the values obtained from DMSO-treated cells (Fig6B). While G418 induced the expected ˜fivefold increase in readthrough, 5-azacytidine failed to induce readthrough while specifically upregulating the expression of endogenous NMD targets in the same cells (Fig6C). These data indicate that 5-azacytidine does not exert its effect on NMD by inducing translational readthrough and that it therefore inhibits NMD by a novel mechanism.

### The inhibition of NMD by 5-azacytidine depends on the induction of MYC

**Figure 7:**
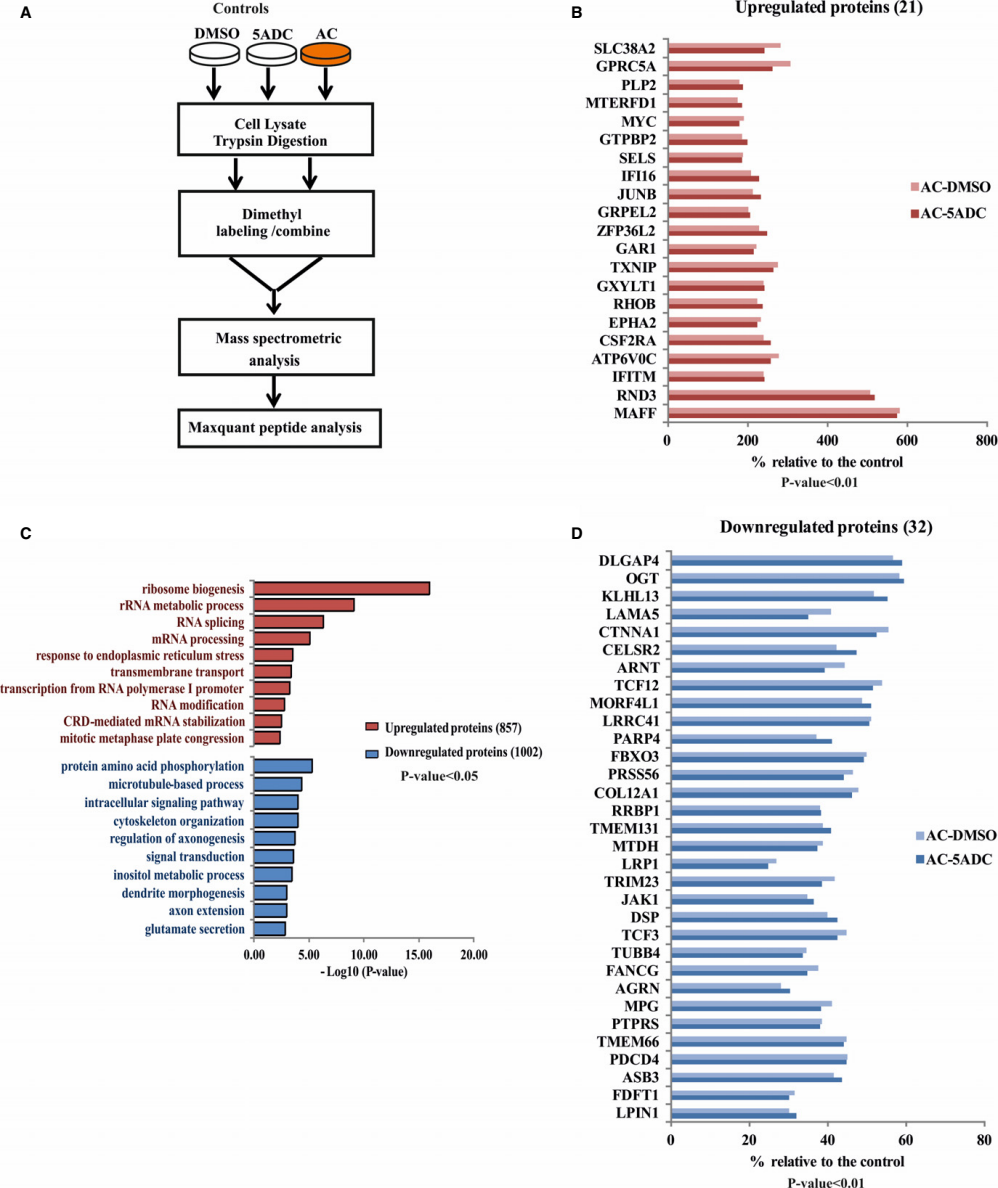
Quantitative mass spectrometry reveals candidate proteins including MYC to be induced by 5-azacytidine treatment Schematic representation of experimental setup followed for quantitative mass spectrometric analysis.Graphical representation of gene ontology studies performed on the proteins upregulated or downregulated upon 5-azacytidine treatment with a *P*-value < 0.05.Bar diagram showing genes upregulated upon 5-azacytidine treatment with a *P*-value < 0.01. Data represents the average of two replicates.Bar diagram showing the list of genes downregulated upon 5-azacytidine treatment with value < 0.01. Data represent the average of two replicates.

**Figure 8:**
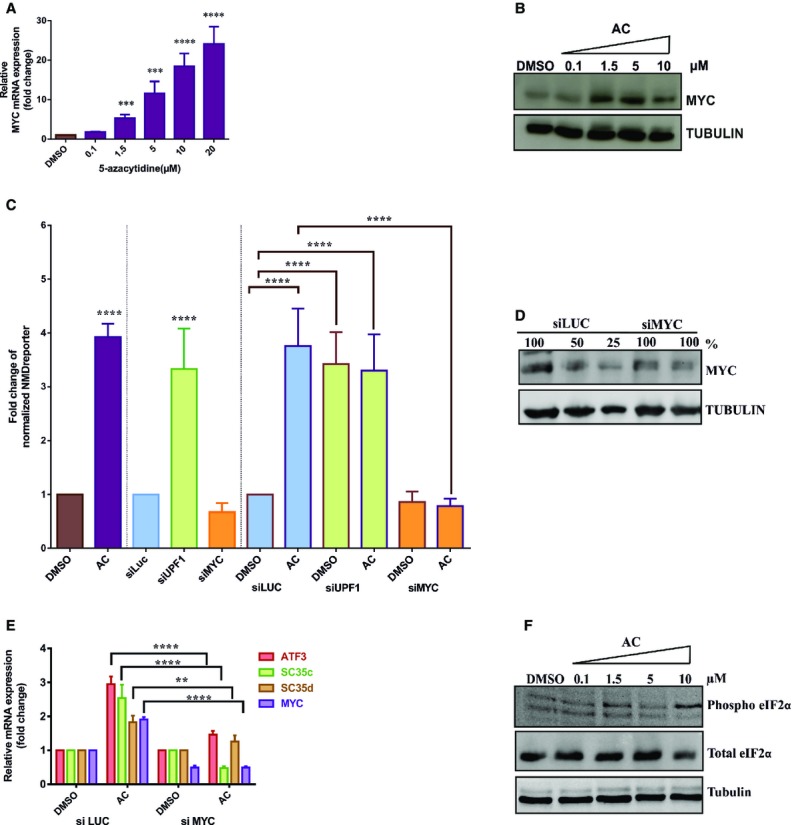
NMD inhibition by 5-azacytidine depends on the stimulation of MYC MYC qRT-PCR analysis of total cellular RNA following treatment with increasing concentrations of 5-azacytidine or DMSO, a negative control. GAPDH mRNA is used for normalization. One-way ANOVA followed by Holm–Sidak multiple comparisons test was performed to analyze the significance, *N* = 3 and ***P = 0.0002 and ***P = 0.0006 for 5-azacytidine (1.5 and 5 μM) and ****P < 0.0001 for 5-azacytidine (10 and 20 μM), respectively. Each bar represents average ± SD.Western blot of HeLa cell lysates following treatment with either DMSO as a negative control, or increasing doses of 5-azacytidine (AC) (0.1, 1.5, 5 and 10 μM) for 18 h and staining with MYC-specific antibodies. Tubulin was used as a loading control.Reporter luciferase activity following treatment with either DMSO or 1.56 μM AC, siLUC, siUPF or siMYC or with combined treatment of siLUC, siUPF and siMYC with DMSO or 1.56 μM AC. The *x*-axis shows the treatment used, and the *y*-axis shows the fold change in comparison to the cells treated with the DMSO or siLUC controls. For single siRNA treatments, one-way ANOVA followed by Holm–Sidak multiple comparisons test was performed to analyze the significance, *N* = 8 and ****P < 0.0001 for siUPF. For DMSO and AC treatment, Student′s *t*-test was performed to analyze the significance, *N *= 3 and ****P < 0.0001 for AC, and for combined treatment, two-way ANOVA followed by Newman–Keuls multiple comparison test was performed to analyze the significance, *N* = 12 and ****P < 0.0001 for AC (si LUC), DMSO, AC (siUPF) and AC (siMYC). Each bar represents average ± SD.Western blot of HeLa cells following treatment with either siLUC as negative control or with siMYC. Tubulin was used as a loading control. Undiluted (100%) or diluted (50 and 25%) lysates of siLUC-treated cells were used for semi-quantification.qRT-PCR analysis of the endogenous NMD targets ATF3, SC35c, SC35d and MYC following treatment of HeLa cells with siLUC or siMYC combined with DMSO or 5-azacytidine (AC). The fold change on the *y*-axis represents the relative quantification of transcripts versus GAPDH mRNA, which is used as a normalization control. The signal detected in siLUC + DMSO-treated cells is set as 1. Two-way ANOVA followed by Newman–Keuls multiple comparison test was performed to analyze the significance. *N* = 3 and **P = 0.0022 for SC35d and ****P < 0.0001 for ATF3, SC35c and MYC. Each bar represents average ± SD.Western blot of HeLa cell lysates following treatment with either DMSO as a negative control, or increasing doses of 5-azacytidine (AC) (0.1, 1.5, 5 and 10 μM) for 18 h and staining with antibodies that specifically detect phospho eIF2α and total eIF2α. Tubulin was used as a loading control. Source data are available online for this figure.

In order to reveal the inhibitory mechanism of 5-azacytidine on NMD, we performed a semi-quantitative global mass spectrometry analysis (Boersema *et al*, 2009). We treated HeLa cells either with 5-azacytidine or with DMSO or 5-aza-2′-deoxycytidine as negative controls for 18 h and labeled proteomic lysates with dimethyl light or intermediate isotopes before subjecting the samples to mass spectrometry (Fig7A). This analysis revealed 857 proteins to be upregulated and 1,002 proteins to be downregulated upon 5-azacytidine treatment with a *P-*value of < 0.05 (Fig7B). Gene ontology studies showed proteins involved in ribosomal biogenesis and in RNA processing to be among the most significantly increased polypeptides, and kinases involved in amino acid phosphorylation to be the most significantly downregulated group upon 5-azacytidine treatment (Fig7B). When the *P*-value is limited to < 0.01, 21 proteins are identified to be upregulated and 32 proteins to be downregulated by 5-azacytidine (Fig7C and D). Among the most strongly increased proteins is MYC, which has previously been reported to inhibit NMD in B-lymphocyte differentiation (Wang *et al*, 2011). We validated increased MYC expression at the mRNA and protein levels in HeLa cells treated with increasing doses of 5-azacytidine (Fig8A and 8B). Immunoblot analysis of lysates from 5-azacytidine-treated cells shows upregulation of MYC in a dose-dependent manner (Fig8B).

We next tested whether the correlation between the upregulation of MYC and the down-modulation of NMD efficiency by 5-azacytidine is causally related. We thus depleted MYC from cells expressing the NMD reporter by specific siRNA treatment or treated the cells with luciferase siRNAs as a negative control. The efficiency of the MYC ablation was monitored by immunoblotting and qRT-PCR analysis, showing depletion of both the mRNA and the protein to approximately 50% (Fig8D and E). Depletion of the essential NMD factor UPF1 and treatment of cells with 5-azacytidine alone served as positive controls. As expected, the NMD reporter luciferase activity increased threefold to fourfold following 5-azacytidine treatment or UPF1 depletion. This effect is specifically lost in cells depleted of MYC (Fig8C), implicating MYC as necessary, causal component of the cellular pathway resulting in the inhibition of NMD following azacytidine treatment. Notably, depletion of UPF1 and treatment of the cells with 5-azacytidine appear not to be synergistic, indicating that both interventions likely affect the same pathway.

We also analyzed the endogenous NMD targets ATF3, SC35C, SC35D as well as MYC by qRT-PCR. RNA was isolated from cells exposed to MYC-specific siRNAs (or siLUC as a negative control) and subsequently treated with 5-azacytidine or with vehicle control. All of the three tested endogenous NMD targets and MYC RNA show approximately 1.5–threefold increase in expression following treatment of the cells with 5-azacytidine and the control siLUC. In MYC-depleted cells, the effect of 5-azacytidine on the endogenous targets is lost, further confirming the importance of MYC for the effect of 5-azacytidine on NMD (Fig8E). It has previously been reported that the effect of MYC on NMD results from the increased phosphorylation of eIF2α (Wang *et al*, 2011). We thus analyzed eIF2α phosphorylation following treatment of the cells with increasing concentrations of 5-azacytidine (Fig8F). At the low concentrations of 5-azacytidine that inhibit NMD without toxic effects, the drug does not induce eIF2α phosphorylation, which is only noticed at higher, toxic concentrations. Consistent with the results shown in Fig5, these data show that eIF2α phosphorylation (which typically inhibits translation) does not explain the effects of 5-azacytidine on NMD.

In summary, these data demonstrate that the inhibitory effect of 5-azacytidine on NMD requires MYC expression.

## Discussion

The identification of 5-azacytidine as a potent and specific inhibitor of NMD represents one of the key important novel findings of the analyses reported here. Our results show that 5-azacytidine does not act by interfering with one of the known mechanisms that are important for efficient NMD such as active translation, correct translation termination at the PTC, or the abundance of NMD co-factors. Quantitative global mass spectrometric analysis implicated MYC as a protein involved in the response to 5-azacytidine, and this protein was recently shown to inhibit NMD when overexpressed in B lymphocytes (Wang *et al*, 2011). Here, we find that depleting only approximately 50% of MYC expression almost completely blocks the effect of 5-azacytidine on NMD efficiency.

Presently, it remains an open question as to how 5-azacytidine upregulates MYC and how MYC expression inhibits NMD. These effects are unlikely to be mediated via the known inhibitory effect of 5-azacytidine on DNA methylation, because 5-aza-2′-deoxycytidine, a substance that shares this DNA-demethylating property (Stresemann & Lyko, 2008), fails to inhibit NMD (see Table1 and Fig2). However, unlike 5-aza-2′-deoxycytidine, 5-azacytidine interferes with RNA methylation (Glazer & Peale, 1979; Schaefer *et al*, 2009), which could play a role in its NMD inhibitory activity. The analysis of the role of RNA methyltransferases could be an important next step in studying the mechanism of NMD inhibition by 5-azacytidine.

MYC is a multi-functional protein that controls cell growth, proliferation and apoptosis by stimulating or inhibiting the transcription of a large number of genes (Levens, 2002). It also causes post-translational modifications of proteins (Secombe & Eisenman, 2007), stimulates translation (Barna *et al*, 2008) and plays a role in DNA replication (Dominguez-Sola *et al*, 2007). Furthermore, MYC has recently been shown to broadly affect microRNA expression (Chang *et al*, 2008) and specifically to induce miR-128, which was shown to upregulate NMD targets (Bruno *et al*, 2011). Although the mechanistic link between NMD and MYC requires intensive additional work, 5-azacytidine and increased MYC expression might inhibit NMD via altered microRNA expression.

The identification of 5-azacytidine as an NMD inhibitor may well be highly relevant from a clinical perspective, because this drug is already approved for the treatment of chronic diseases such as myelodysplastic syndrome and chronic myelomonocytic leukemia (Gryn *et al*, 2002; Sullivan *et al*, 2005; Keating, 2012). Importantly, the concentration of 5-azacytidine required for NMD inhibition in cells is similar or even below the drug levels in the plasma of patients undergoing anti-leukemic therapy (Stresemann & Lyko, 2008). In contrast to other known but more toxic NMD inhibitors (Keeling & Bedwell, 2011), the modest clinical toxicity of 5-azacytidine at these doses justifies its exploration for the treatment of life-threatening diseases that would benefit from NMD inhibition and an increased expression of PTC-mutated transcripts (Bhuvanagiri *et al*, 2010). Such PTC-mutated transcripts encode C-terminally truncated proteins, which are (partially) functional, and NMD inhibition can thus result in a therapeutic effect. Some forms of Duchenne muscular dystrophy and cystic fibrosis, which are caused by PTC mutations in the 3′ region of the dystrophin and the CFTR genes, respectively, exemplify diseases that may benefit from such an approach (Linde & Kerem, 2008; Keeling & Bedwell, 2011). Similarly, some forms of cancer such as gastric cancer, lung cancer and T-cell prolymphocytic leukemia that are driven by PTC mutations in tumor suppressor genes including CDH1, p53 and CDKN1B may also be considered for treatment with an NMD inhibitor (Lehman *et al*, 1991; Karam *et al*, 2008; Metzeler *et al*, 2011). When considering 5-azacytidine as a potential drug for the treatment of NMD-related disorders, it must also be considered that MYC represents a potent oncogene that is upregulated in many forms of cancer (Yokota *et al*, 1986) and which can induce tumor formation in transgenic animals (Langenau *et al*, 2003; Shachaf *et al*, 2004). This oncogenic potential may limit the long-term usefulness of this drug for the treatment of non-malignant disorders.

We would expect that 5-azacytidine acts synergistically with compounds that induce translational readthrough at premature termination codons (Martin *et al*, 2014). The mechanistic principle of such compounds relies on the accommodation of a near-cognate tRNA at the stop codon, thus functionally converting a PTC into a missense mutation (Burke & Mogg, 1985; Bhuvanagiri *et al*, 2010). The concept of this approach has been proven to be effective in cystic fibrosis and in Duchenne muscular dystrophy (Linde & Kerem, 2008; Keeling *et al*, 2012). Specifically, nonsense suppression has been found to be more effective in patients with naturally less efficient NMD than in those with more efficient NMD (Linde *et al*, 2007a). NMD inhibitors such as 5-azacytidine may thus increase the abundance of mRNA substrates that would be targeted by compounds that are currently being developed for the induction of translational readthrough (Lee & Dougherty, 2012).

In conclusion, we demonstrate that 5-azacytidine modulates NMD efficiency in a MYC-dependent manner. Our data suggest a clinical potential of this drug for the treatment of Mendelian and acquired genetic diseases that are caused by PTC mutations, and hence its potential to advance personalized medicine.

## Materials and Methods

### Cell culture, siRNA transfections and plasmid transfection

HeLa cells stably expressing either HBB PTC-mutant or wild-type renilla luciferase reporter constructs were used in this study (Boelz *et al*, 2006). Stably transfected cell lines were cultivated in DMEM medium (Invitrogen) supplemented with 10% fetal calf serum (FCS) and 1% penicillin–streptomycin (PS) and treated with 1 μg/ml doxycycline for induction of expression of the PTC-mutant or wild-type reporters.

For siRNA-mediated knockdown experiments, 6-well plates were seeded with 1 × 10^5^ HeLa cells expressing the PTC reporter in the morning of day 1. Four to 6 h later, transient transfection of siRNA was performed according to the manufacturer's recommendations using 10 μl siRNAs (20 μM stock) and 3 μl Oligofectamine reagent (Invitrogen) in Opti-MEM medium (Invitrogen) without serum and antibiotics. On day 2, 2 ml of fresh DMEM medium with 10% FCS, 1% PS and 1 μg/ml doxycycline was added to the cells. Seventy-two hours after siRNA transfection, cells were lysed (Promega, E291A) and luciferase activity was determined using the renilla luciferase assay reagents (Promega, E2820).

For testing luciferase activity of known NMD inhibitors, 6-well plates were seeded with 2 × 10^5^ HeLa cells expressing the PTC reporter in DMEM medium with 10% FCS, 1% PS and 1 μg/ml doxycycline, the day after cells were treated with equal amounts of DMSO or 0.5 μg/ml of anisomycin, and 40 μg/ml of cycloheximide. Eighteen hours later, cells were harvested and lysed with 300 μl of buffer (Promega E291A) and luciferase activity was determined using the renilla luciferase assay reagents (Promega, E2820).

Calu-6 cells were grown in RPMI medium containing 10% FCS and 1% PS. 2 × 10^5^ Calu-6 cells were seeded in a 6-well plate and treated with five different dilutions (0.5, 1, 3, 5 and 10 μM) of 5-azacytidine. DMSO was used as a negative control. Cells were harvested after 18 h of treatment and collected in 300 μl of RNA lysis buffer (RLT, Qiagen), and RNA was isolated according to the QiagenRNeasy protocol (Qiagen, 74106). One microgram of total cytoplasmic RNA was reverse transcribed following the first-strand cDNA synthesis according to the protocol of RevertAid™ H Minus Reverse Transcriptase (Thermo scientific, EP0451) and used for qPCR analysis.

For readthrough assays, HeLa cells were transfected with 2 μg of either p2luc-wild-type or p2luc-TAG plasmids (Ivanov *et al*, 2008). On day 2, the cells were treated with 1.56 μM of 5-azacytidine or 600 μM of G418. Forty-eight hours after transfection, cells were harvested and lysed with 300 μl passive lysis buffer (Promega, E1941) and luciferase activity was determined using the dual luciferase reporter assay (Promega, E1910) as described in Ivanov *et al* (2008). For RNA isolation from the same sample, a threefold dilution of RNA lysis buffer (RLT, Qiagen) was added to 150 μl of the sample and RNA isolation was performed according to the Qiagen RNeasy protocol (Qiagen, 74106).

### Compound libraries and preparation

A total of 1120 compounds were obtained from the Prestwick Chemical Library® (Prestwick Chemical, Washington, DC) and selected for screening of potential NMD modulators. All the compounds were stored at 2 mg/ml in 100% DMSO, and the compounds were tested at final concentrations of 5 and 0.1 μg/ml for 18 h in 0.25% DMSO. All the compounds were dispensed with the Evolution P3 pipetting platform (Perkin Elmer). After 18 h, 20 μl of Renilla-Glo™ luciferase assay reagent (Promega, E2750) was added to all wells using a Flex Drop IV EXi reagent dispenser (Perkin Elmer). The luminescence signal was read out 10 min later on an Envision plate reader with ultrasensitive luminescence detector (Perkin Elmer). The Renilla-Glo™ reagent lyses the cells and generates a luminescent signal, which is proportional to the expression of the NMD reporter. Two controls were used on each plate of cells: (i) cells treated with anisomycin (positive control) and (ii) cells in media containing 0.25% DMSO (negative control).

### High-throughput screening and toxicity measurements

HeLa cells (˜3 × 10^3^) stably expressing the PTC reporter were seeded in 384-well culture plates with Flex Drop IV EXi reagent dispenser (Perkin Elmer) a day prior to treatment in 40 μl media with 1 μg/ml doxycycline. DMEM without phenol red with 10% FCS and 1% PS was used throughout the screening. The following day, cells were treated with compounds from the Prestwick Chemical Library®. After 18 h, cells were harvested and lysed with the Renilla-Glo™ luciferase assay system (Promega, E2750). The luminescence signal was detected in a plate reader after 10 min. Along with the top two hits 5-azacytidine and lycorine, compounds which showed > 150% upregulation of the PTC reporter with respect to the negative control were also selected for secondary screening using HeLa cells expressing a wild-type HBB renilla luciferase reporter. In total, six compounds were selected for secondary screening (5-azacytidine, lycorine, emetine, cephaeline, mebendazole, nocodazole).

For dose–response studies with the selected hits, 384-well plates were seeded as before with HeLa cells (˜3 × 10^3^) stably expressing wild-type or mutant HBB renilla luciferase reporters a day prior to treatment in 40 μl media with 1 μg/ml doxycycline. DMEM without phenol red (Life Technologies) with 10% FCS and 1% PS was used. The day after, cells were treated with a serial dilution ranging from 0.0049 to 50 μM of 5-azacytidine, lycorine, emetine, cephaeline, mebendazole or nocodazole.

For the testing of nucleoside and nucleotide analogues, 384-well plates were seeded with wild-type or mutant HBB reporter-expressing HeLa cells in DMEM medium with 10% FCS, 1% PS and 1 μg/ml doxycycline. The day after, cells were treated with a serial dilution ranging from 0.0049 to 50 μM of each compound mentioned in Table1. Cell toxicity of all compounds was analyzed by performing cell viability assay in parallel with a serial dilution of 0.0065–50 μM. The ATPLite 1step kit (Perkin Elmer, 6016739) was used to detect the ATP from the cells which is directly proportional to cell viability (Crouch *et al*, 1993).

### *Z*′ factor for pass/fail criterion

To calculate uniformity from plate to plate and from screening batch to batch, the *Z*′ value was calculated for high-throughput screening (Iversen *et al*, 2006). The *Z*′ factor compares the baseline background (minimum renilla luciferase signal) from the DMSO negative control, and the maximum signal of the positive control anisomycin. *Z*′ factor = 1 – (3× (standard deviation anisomycin + standard deviation of DMSO)/(average anisomycin – average DMSO)). The average *Z*′-value of the screen with 5 μg/ml was 0.65, and the average *Z*′-value of the screen with 0.1 μg/ml was 0.70, which indicates that the screening was accurate and robust.

### RNA and western blot analysis

6-well plates were seeded with 2 × 10^5^ HeLa cells expressing either the wild-type or the PTC-mutated HBB reporter in DMEM medium with 10% FCS, 1% PS and 1 μg/ml doxycycline. On day 2, cells were treated with equal amounts of either DMSO as a negative control, 0.5 μg/ml of anisomycin or 40 μg/ml of cycloheximide as positive controls, 0.75 or 1.56 μM of 5-azacytidine, or 1.56 μM of 5-aza-2′-deoxycytidine. Eighteen hours later, cells were harvested in 1 ml of Trizol (Sigma, T3934), and 1.5–5 μg of total cytoplasmic RNA was analyzed by Northern blot analysis with a β-globin-specific radiolabeled antisense DNA probe as described before (Thermann *et al*, 1998). For normalization, the membrane was reprobed with a GAPDH probe. Subsequently, the ratio between the normalized mRNA level transcribed from the PTC-mutated and the wild-type constructs following NMD inhibition was calculated and compared with this ratio in DMSO-treated cells. Radioactive signals were quantified by phosphor imaging in a FLA-3000 fluorescent image analyser (Raytest, Fujifilm). Mean values and standard deviations of all experiments were calculated from at least three independent experiments. Each batch of 5-azacytidine (Tocris: 3842) used was tested for the downregulation of DNA methyltransferase 1 (DNMT1).

For simultaneous analysis of mRNA and proteins, cells were lysed in a buffer containing protease and phosphatase inhibitors [10 mM Tris–HCl (pH 7.5), 8 mM MgCl_2_, 10 mM NaCl, 1 mM DTT, 0.5% NP-40, 1% sodium deoxycholate, complete protease inhibitor and phosphatase inhibitor (Roche Applied Science)], and aliquots for protein analysis were taken before RNA isolation. Western blot analysis was then performed using 20–30 μg of cell lysate. Subsequently, proteins were transferred to PVDF membranes using a semi-dry electro blotting system. Membranes were blocked with 5% non-fat skimmed milk in TBS-Tween (0.1%), and the membrane was probed with antibodies directed against UPF1 (Bethyl, A301-902A), UPF2 [kindly provided by Jens Lykke-Andersen (Lykke-Andersen *et al*, 2000)], UPF3A (Sigma, SAB1402625), UPF3B (Sigma, SAB2102656), MAGOH (abcam, ab38768), Y14 (Immunoquest, IQ220), BTZ (Abcam, ab118803), RNPS1 (Santa Cruz, sc-19940), SMG1 (Calbiochem, DR1035), SMG7 (Bethyl, A302-170A), and phosphorylated UPF1 [kindly provided by Akio Yamashita (Okada-Katsuhata *et al*, 2012)], MYC (Sigma-Aldrich, M5546), eIF2α (Cell Signalling, 2103) and Phospho eIF2α (Cell Signalling, 3597).

### ^35^S metabolic labeling studies

2 × 10^5^ HeLa cells were seeded in six-well plates on day 1. Culture medium (DMEM with 10% FCS, 1% PS) was changed on day 2. On day 3, HeLa cells were treated with equal amounts of either DMSO or 1.56 μM of 5-aza-2′-deoxycytidine as negative controls, 0.5 μg/ml of anisomycin or 40 μg/ml of cycloheximide as positive controls and 1.56 μM of 5-azacytidine. Eighteen hours later, the cells were washed and incubated for 20 min in methionine- and cysteine-free medium (10% FCS, 1% PS and 1 μg/ml glutamine) and then incubated for 2 h in the restriction medium supplemented with ^35^S-methionine and ^35^S-cysteine (10 mCi/ml), and the respective chemical treatment (DMSO or 1.56 μM of 5-aza-2′-deoxycytidine or 0.4 μg/ml of anisomycin 40 μg/ml of cycloheximide or 1.56 μM of 5-azacytidine) was readded to the restricted culture medium. After 2 h, cells were washed twice in ice-cold PBS, harvested in 200 μl RIPA buffer (50 mM Tris–HCl at pH 7.5, 150 mM CaCl_2_, 1% NP-40, 0.5% sodium deoxycholate, 0.1% SDS) (Banihashemi *et al*, 2006) and centrifuged for 5 min at 15,871 *g* at 4°C. Ten microlitre of the supernatant was spotted onto a glass microfiber filter (Whatmann), dried, washed with ice-cold 15% TCA and incubated on ice for 30 min. A second washing step followed with ice-cold 15% TCA and two wash steps with ice-cold 100% ethanol was carried out. Radioactivity was then determined by scintillation counting. For gel analysis, 15 μg of lysate was loaded on 10% SDS gel and radioactive signals were quantified by phosphor imaging in a FLA-3000 fluorescent image analyser (Raytest, Fujifilm). Coomassie stained gels were used as loading controls.

### Polysomal profile analysis

HeLa cells were treated with DMSO, arsenite (300 μM, 2 h) or 5-azacytidine (1.56 μM, or 10 μM, 18 h). Shortly, before lysis, cells are treated for 10 min with cycloheximide 100 μg/ml and then washed with cold PBS containing 100 μg/ml cycloheximide. Cells were then harvested and lysed in 0.2 ml of lysis buffer containing 15 mM Tris, pH 7.4, 15 mM MgCl_2_, 300 mM NaCl, 1% Triton X-100, 100 μg/ml cycloheximide, 500 μg/ml heparin, 0.2 U/ml RNasin, 0.1% 2-mercaptoethanol and EDTA-free protease inhibitor. Cell lysates were centrifugation at 9,391 *g* for 10 min at 4°C. Supernatants were loaded onto the 17.5–50% sucrose gradients and centrifuged in a SW60 rotor at 164,756 *g* for 2.5 h at 4°C. Fractions were then eluted from the top of the gradient using a Teledyne Isco (Lincoln, NE) gradient elution system (Hofmann *et al*, 2012). Polysomal profiles were obtained by measuring absorbance at 254 nm.

### Quantitative real-time PCR

RNA was isolated by using the QiagenRNeasy method (Qiagen, cat. no 74106). One microgram of total cytoplasmic RNA was reverse transcribed following first-strand cDNA synthesis protocol of RevertAid™ H Minus Reverse Transcriptase (Thermo scientific, EP0451). The RT–PCR was performed on StepOnePlus™ machine (Applied Biosystems), using Absolute SYBR green mix (Thermo scientific, AB-1158/A). Primers for the NMD sensitive RPL3 variant were described previously (Cuccurese *et al*, 2005). Other primers used in this study are mentioned below: SC35c-forward: 5′GGCGTGTATTGGAGCAGATGTA-3′; reverse: 5′- CTGCTACACAACTGCGCCTTTT-3′; SC35d-forward: 5′- CGGTGTCCTCTTAAGAAAATGATGTA-3′; reverse: 5′-CTGCTACACAACTGCGCCTTTT-3′; SC35a NMD-insensitive control forward: 5′-CGTGCCTGAAACTGAAACCA-3′; reverse 5′-TTGCCAACTGAGGCAAAGC-3′, P53-forward: 5′-GAGGTTGGCTCTGACTGTACC-3′; reverse: 5′-TCCGTCCCAGTAGATTACCAC-3′; GAPDH-forward: 5′-TGAGCTTGACAAAGTGGTCG-3′; reverse: 5′-GGCTCTCCAGAACATCATCC-3′; RPL13 NMD-insensitive control forward: 5′-CTCTCAAGGTGTTTGACGGC-3′; reverse: 5′-TTTATTGGGCTCAGACCAGG-3′; ATF3-forward: 5′-GCCATTGGAGAGCTGTCTTC-3′; reverse: 5′-GGGCCATCTGGAACATAAGA-3′; preHBB-forward: 5′-CAGCTACAATCCAGCTACC-3′; reverse: 5′-CACTTTTCTGATAGGCAGC-3′; preGAPDH-forward: 5′-AGGGCCCTGACAACTCTTTT-3′; reverse: 5′-AGGGGTCTACATGGCAACTG-3′; MYC- forward: 5′-AAACACAAACTTGAACAGCTAC-3′; MYC-reverse: 5′-ATTTGAGGCAGTTTACATTATGG-3′.

### Dimethyl labeling and quantitative mass spectrometry

Cells were treated with DMSO, 5-azacytidine or 5-aza-2′-deoxycytidine for 18 h and then harvested in 1 ml PBS. After lysis of cells in 0.1% RapiGest (Waters) and 50 mM (NH_4_) HCO_3_, extracted proteins were subsequently reduced and alkylated with 5 mM DTT and 10 mM iodoacetamide and digested overnight with sequencing-grade modified trypsin (Promega). Peptides were labeled on SepPak C18 cartridges (Waters) with labeling reagent (light and intermediate, with CH_2_O (Fisher) plus NaBH_3_CN (Fluka) or CD_2_O (Isotec) plus NaBH_3_CN, respectively) as described in Boersema *et al* (2009).

Peptides were separated using the nano ACQUITY UPLC system (Waters) fitted with a trapping column [nanoAcquity Symmetry C18; 5 μm (average particle diameter); 180 μm (inner diameter) × 20 mm (length)] and an analytical column [nanoAcquity BEH C18; 1.7 μm (average particle diameter); 75 μm (inner diameter) × 200 mm (length)]. Peptides were separated on a 120-min gradient and were analyzed by electrospray ionization–tandem mass spectrometry on an OrbitrapVelos Pro (Thermo Fisher Scientific). Raw data files of mass spectrometry were processed with the MaxQuant quantitative proteomics software package (version 1.3.0.5) (Cox & Mann, 2008). The Andromeda search engine (version 1.3.0.5) of MaxQuant was used to search the derived peak list using the human database Universal Protein Resource Knowledge base (2012.07.11).

### Statistical analysis

All data represent average ± SD. Data were analyzed by one-way ANOVA followed by Holm–Sidak multiple comparisons test or two-way ANOVA followed by Newman–Keuls multiple comparison test or by Student's *t*-test, as appropriate with GraphPad Prism v.6 software.

## Supporting Information

for this article is available online: http://embomolmed.embopress.org

Supplementary Figures S1–S3

Review Process File

## References

[b1] Banihashemi L, Wilson GM, Das N, Brewer G (2006). Upf1/Upf2 regulation of 3′ untranslated region splice variants of AUF1 links
nonsense-mediated and A+U-rich element-mediated mRNA decay. Mol Cell Biol.

[b2] Barna M, Pusic A, Zollo O, Costa M, Kondrashov N, Rego E, Rao PH, Ruggero D (2008). Suppression of Myc oncogenic activity by ribosomal protein
haploinsufficiency. Nature.

[b3] Belgrader P, Cheng J, Maquat LE (1993). Evidence to implicate translation by ribosomes in the mechanism by which nonsense
codons reduce the nuclear level of human triosephosphate isomerase mRNA. Proc Natl Acad Sci USA.

[b4] Bhuvanagiri M, Schlitter AM, Hentze MW, Kulozik AE (2010). NMD: RNA biology meets human genetic medicine. Biochem J.

[b5] Boelz S, Neu-Yilik G, Gehring NH, Hentze MW, Kulozik AE (2006). A chemiluminescence-based reporter system to monitor nonsense-mediated mRNA
decay. Biochem Biophys Res Commun.

[b6] Boersema PJ, Raijmakers R, Lemeer S, Mohammed S, Heck AJ (2009). Multiplex peptide stable isotope dimethyl labeling for quantitative
proteomics. Nat Protoc.

[b7] Bruno IG, Karam R, Huang L, Bhardwaj A, Lou CH, Shum EY, Song HW, Corbett MA, Gifford WD, Gecz J (2011). Identification of a microRNA that activates gene expression by repressing
nonsense-mediated RNA decay. Mol Cell.

[b8] Burke JF, Mogg AE (1985). Suppression of a nonsense mutation in mammalian cells *in vivo* by the
aminoglycoside antibiotics G-418 and paromomycin. Nucleic Acids Res.

[b9] Carter MS, Doskow J, Morris P, Li S, Nhim RP, Sandstedt S, Wilkinson MF (1995). A regulatory mechanism that detects premature nonsense codons in T-cell receptor
transcripts *in vivo* is reversed by protein synthesis inhibitors *in
vitro*. J Biol Chem.

[b10] Chang YF, Imam JS, Wilkinson MF (2007). The nonsense-mediated decay RNA surveillance pathway. Annu Rev Biochem.

[b11] Chang TC, Yu D, Lee YS, Wentzel EA, Arking DE, West KM, Dang CV, Thomas-Tikhonenko A, Mendell JT (2008). Widespread microRNA repression by Myc contributes to tumorigenesis. Nat Genet.

[b12] Christman JK (2002). 5-Azacytidine and 5-aza-2′-deoxycytidine as inhibitors of DNA methylation:
mechanistic studies and their implications for cancer therapy. Oncogene.

[b13] Cox J, Mann M (2008). MaxQuant enables high peptide identification rates, individualized p.p.b.-range mass
accuracies and proteome-wide protein quantification. Nat Biotechnol.

[b14] Crouch SP, Kozlowski R, Slater KJ, Fletcher J (1993). The use of ATP bioluminescence as a measure of cell proliferation and
cytotoxicity. J Immunol Methods.

[b15] Cuccurese M, Russo G, Russo A, Pietropaolo C (2005). Alternative splicing and nonsense-mediated mRNA decay regulate mammalian ribosomal
gene expression. Nucleic Acids Res.

[b16] Dang Y, Low WK, Xu J, Gehring NH, Dietz HC, Romo D, Liu JO (2009). Inhibition of nonsense-mediated mRNA decay by the natural product pateamine A through
eukaryotic initiation factor 4AIII. J Biol Chem.

[b17] Dominguez-Sola D, Ying CY, Grandori C, Ruggiero L, Chen B, Li M, Galloway DA, Gu W, Gautier J, Dalla-Favera R (2007). Non-transcriptional control of DNA replication by c-Myc. Nature.

[b18] Dranchak PK, Di Pietro E, Snowden A, Oesch N, Braverman NE, Steinberg SJ, Hacia JG (2011). Nonsense suppressor therapies rescue peroxisome lipid metabolism and assembly in
cells from patients with specific PEX gene mutations. J Cell Biochem.

[b19] Finkel RS, Flanigan KM, Wong B, Bonnemann C, Sampson J, Sweeney HL, Reha A, Northcutt VJ, Elfring G, Barth J (2013). Phase 2a study of ataluren-mediated dystrophin production in patients with nonsense
mutation Duchenne muscular dystrophy. PLoS ONE.

[b20] Gehring NH, Kunz JB, Neu-Yilik G, Breit S, Viegas MH, Hentze MW, Kulozik AE (2005). Exon-junction complex components specify distinct routes of nonsense-mediated mRNA
decay with differential cofactor requirements. Mol Cell.

[b21] Glazer RI, Peale AL (1979). Evidence that xylosyladenine affects methylation by inhibition of
S-adenosyl-L-methionine synthesis. Cancer Lett.

[b22] Gonzalez-Hilarion S, Beghyn T, Jia J, Debreuck N, Berte G, Mamchaoui K, Mouly V, Gruenert DC, Deprez B, Lejeune F (2012). Rescue of nonsense mutations by amlexanox in human cells. Orphanet J Rare Dis.

[b23] Gryn J, Zeigler ZR, Shadduck RK, Lister J, Raymond JM, Sbeitan I, Srodes C, Meisner D, Evans C (2002). Treatment of myelodysplastic syndromes with 5-azacytidine. Leuk Res.

[b24] Guthrie OW (2008). Aminoglycoside induced ototoxicity. Toxicology.

[b25] Hentze MW, Kulozik AE (1999). A perfect message: RNA surveillance and nonsense-mediated decay. Cell.

[b26] Hofmann S, Cherkasova V, Bankhead P, Bukau B, Stoecklin G (2012). Translation suppression promotes stress granule formation and cell survival in
response to cold shock. Mol Biol Cell.

[b27] Holbrook JA, Neu-Yilik G, Hentze MW, Kulozik AE (2004). Nonsense-mediated decay approaches the clinic. Nat Genet.

[b28] Holbrook JA, Neu-Yilik G, Gehring NH, Kulozik AE, Hentze MW (2006). Internal ribosome entry sequence-mediated translation initiation triggers
nonsense-mediated decay. EMBO Rep.

[b29] Isken O, Maquat LE (2008). The multiple lives of NMD factors: balancing roles in gene and genome
regulation. Nat Rev Genet.

[b30] Ivanov PV, Gehring NH, Kunz JB, Hentze MW, Kulozik AE (2008). Interactions between UPF1, eRFs, PABP and the exon junction complex suggest an
integrated model for mammalian NMD pathways. EMBO J.

[b31] Iversen PW, Eastwood BJ, Sittampalam GS, Cox KL (2006). A comparison of assay performance measures in screening assays: signal window,
Z′ factor, and assay variability ratio. J Biomol Screen.

[b32] Karam R, Carvalho J, Bruno I, Graziadio C, Senz J, Huntsman D, Carneiro F, Seruca R, Wilkinson MF, Oliveira C (2008). The NMD mRNA surveillance pathway downregulates aberrant E-cadherin transcripts in
gastric cancer cells and in CDH1 mutation carriers. Oncogene.

[b33] Karam R, Wengrod J, Gardner LB, Wilkinson MF (2013). Regulation of nonsense-mediated mRNA decay: implications for physiology and
disease. Biochim Biophys Acta.

[b34] Kashima I, Yamashita A, Izumi N, Kataoka N, Morishita R, Hoshino S, Ohno M, Dreyfuss G, Ohno S (2006). Binding of a novel SMG-1-Upf1-eRF1-eRF3 complex (SURF) to the exon junction complex
triggers Upf1 phosphorylation and nonsense-mediated mRNA decay. Genes Dev.

[b35] Keating GM (2012). Azacitidine: a review of its use in the management of myelodysplastic syndromes/acute
myeloid leukaemia. Drugs.

[b36] Keeling KM, Bedwell DM (2002). Clinically relevant aminoglycosides can suppress disease-associated premature stop
mutations in the IDUA and P53 cDNAs in a mammalian translation system. J Mol Med.

[b37] Keeling KM, Bedwell DM (2011). Suppression of nonsense mutations as a therapeutic approach to treat genetic
diseases. Wiley Interdiscip Rev RNA.

[b38] Keeling KM, Wang D, Conard SE, Bedwell DM (2012). Suppression of premature termination codons as a therapeutic approach. Crit Rev Biochem Mol Biol.

[b39] Kerem E, Konstan MW, De Boeck K, Accurso FJ, Sermet-Gaudelus I, Wilschanski M, Elborn JS, Melotti P, Bronsveld I, Fajac I (2014). Ataluren for the treatment of nonsense-mutation cystic fibrosis: a randomised,
double-blind, placebo-controlled phase 3 trial. Lancet Respir Med.

[b40] Langenau DM, Traver D, Ferrando AA, Kutok JL, Aster JC, Kanki JP, Lin S, Prochownik E, Trede NS, Zon LI (2003). Myc-induced T cell leukemia in transgenic zebrafish. Science.

[b41] Lee HL, Dougherty JP (2012). Pharmaceutical therapies to recode nonsense mutations in inherited
diseases. Pharmacol Ther.

[b42] Lehman TA, Bennett WP, Metcalf RA, Welsh JA, Ecker J, Modali RV, Ullrich S, Romano JW, Appella E, Testa JR (1991). p53 mutations, ras mutations, and p53-heat shock 70 protein complexes in human lung
carcinoma cell lines. Cancer Res.

[b43] Levens D (2002). Disentangling the MYC web. Proc Natl Acad Sci USA.

[b44] Li S, Leonard D, Wilkinson MF (1997). T cell receptor (TCR) mini-gene mRNA expression regulated by nonsense codons: a
nuclear-associated translation-like mechanism. J Exp Med.

[b45] Linde L, Boelz S, Neu-Yilik G, Kulozik AE, Kerem B (2007a). The efficiency of nonsense-mediated mRNA decay is an inherent character and varies
among different cells. Eur J Hum Genet.

[b46] Linde L, Boelz S, Nissim-Rafinia M, Oren YS, Wilschanski M, Yaacov Y, Virgilis D, Neu-Yilik G, Kulozik AE, Kerem E (2007b). Nonsense-mediated mRNA decay affects nonsense transcript levels and governs response
of cystic fibrosis patients to gentamicin. J Clin Invest.

[b47] Linde L, Kerem B (2008). Introducing sense into nonsense in treatments of human genetic
diseases. Trends Genet.

[b48] Lykke-Andersen J, Shu MD, Steitz JA (2000). Human Upf proteins target an mRNA for nonsense-mediated decay when bound downstream
of a termination codon. Cell.

[b49] Maquat LE (2004). Nonsense-mediated mRNA decay: splicing, translation and mRNP dynamics. Nat Rev Mol Cell Biol.

[b50] Martin L, Grigoryan A, Wang D, Wang J, Breda L, Rivella S, Cardozo T, Gardner LB (2014). Identification and characterization of small molecules that inhibit nonsense-mediated
RNA decay and suppress nonsense p53 mutations. Cancer Res.

[b51] Mendell JT, Sharifi NA, Meyers JL, Martinez-Murillo F, Dietz HC (2004). Nonsense surveillance regulates expression of diverse classes of mammalian
transcripts and mutes genomic noise. Nat Genet.

[b52] Metzeler KH, Maharry K, Radmacher MD, Mrozek K, Margeson D, Becker H, Curfman J, Holland KB, Schwind S, Whitman SP (2011). TET2 mutations improve the new European leukemia net risk classification of acute
myeloid leukemia: a Cancer and Leukemia Group B study. J Clin Oncol.

[b53] Mingeot-Leclercq MP, Tulkens PM (1999). Aminoglycosides: nephrotoxicity. Antimicrob Agents Chemother.

[b54] Okada-Katsuhata Y, Yamashita A, Kutsuzawa K, Izumi N, Hirahara F, Ohno S (2012). N- and C-terminal Upf1 phosphorylations create binding platforms for SMG-6 and SMG-5:
SMG-7 during NMD. Nucleic Acids Res.

[b55] Reichman M, Penman S (1973). The mechanism of inhibition of protein synthesis by 5-azacytidine in HeLa
cells. Biochim Biophys Acta.

[b56] Ronkina N, Menon MB, Schwermann J, Arthur JS, Legault H, Telliez JB, Kayyali US, Nebreda AR, Kotlyarov A, Gaestel M (2011). Stress induced gene expression: a direct role for MAPKAP kinases in transcriptional
activation of immediate early genes. Nucleic Acids Res.

[b57] Schaefer M, Hagemann S, Hanna K, Lyko F (2009). Azacytidine inhibits RNA methylation at DNMT2 target sites in human cancer cell
lines. Cancer Res.

[b58] Secombe J, Eisenman RN (2007). The function and regulation of the JARID1 family of histone H3 lysine 4 demethylases:
the Myc connection. Cell Cycle.

[b59] Shachaf CM, Kopelman AM, Arvanitis C, Karlsson A, Beer S, Mandl S, Bachmann MH, Borowsky AD, Ruebner B, Cardiff RD (2004). MYC inactivation uncovers pluripotent differentiation and tumour dormancy in
hepatocellular cancer. Nature.

[b60] Stresemann C, Lyko F (2008). Modes of action of the DNA methyltransferase inhibitors azacytidine and
decitabine. Int J Cancer.

[b61] Sullivan M, Hahn K, Kolesar JM (2005). Azacitidine: a novel agent for myelodysplastic syndromes. Am J Health Syst Pharm.

[b62] Thermann R, Neu-Yilik G, Deters A, Frede U, Wehr K, Hagemeier C, Hentze MW, Kulozik AE (1998). Binary specification of nonsense codons by splicing and cytoplasmic
translation. EMBO J.

[b63] Usuki F, Yamashita A, Higuchi I, Ohnishi T, Shiraishi T, Osame M, Ohno S (2004). Inhibition of nonsense-mediated mRNA decay rescues the phenotype in Ullrich's
disease. Ann Neurol.

[b64] Wang D, Wengrod J, Gardner LB (2011). Overexpression of the c-myc oncogene inhibits nonsense-mediated RNA decay in B
lymphocytes. J Biol Chem.

[b65] Welch EM, Barton ER, Zhuo J, Tomizawa Y, Friesen WJ, Trifillis P, Paushkin S, Patel M, Trotta CR, Hwang S (2007). PTC124 targets genetic disorders caused by nonsense mutations. Nature.

[b66] Yamashita A, Ohnishi T, Kashima I, Taya Y, Ohno S (2001). Human SMG-1, a novel phosphatidylinositol 3-kinase-related protein kinase, associates
with components of the mRNA surveillance complex and is involved in the regulation of
nonsense-mediated mRNA decay. Genes Dev.

[b67] Yokota J, Tsunetsugu-Yokota Y, Battifora H, Le Fevre C, Cline MJ (1986). Alterations of myc, myb, and rasHa proto-oncogenes in cancers are frequent and show
clinical correlation. Science.

