# Supplementary material for: 5-azacytidine inhibits nonsense-mediated decay in a MYC-dependent fashion
Source: EMBO Mol Med. 2014 Oct 15;6(12):1593–609. doi: 10.15252/emmm.201404461 (PMC4287977; doi:10.15252/emmm.201404461)
Supplement: Supplementary file 1 — Supplementary Figures S1–S3 [file emmm0006-1593-sd1.pdf]

EMBO Molecular Medicine

5-azacytidine inhibits nonsense-mediated decay in a MYC dependent fashion

Madhuri Bhuvanagiri, Joe Lewis, Kerstin Putzker, Jonas P. Becker, Stefan Leicht, Jeroen Krijgsveld, Richa Batra, Brad Turnwald, Bogdan Jovanovic, Christian Hauer, Jana Sieber, Matthias Hentze and Andreas Kulozik

Corresponding author: Corresponding Author
Andreas Kulozik, Dr. Andreas Kulozik University of Heidelberg

Review timeline:

Submission date:	27 November 2013
Editorial Decision:	27 December 2013
Resubmission:	22 July 2014
Editorial Decision:	22 August 2014
Revision received:	02 September 2014
Accepted:	03 September 2014

Transaction Report:

(Note: With the exception of the correction of typographical or spelling errors that could be a source of ambiguity, letters and reports are not edited. The original formatting of letters and referee reports may not be reflected in this compilation.)

Editor: Roberto Buccione

1st Editorial Decision 27 December 2013

Thank you for the submission of your manuscript to EMBO Molecular Medicine. We have now received reports from all three Reviewers.

As you will see, the reviewers point to significant and fundamental issues that, I am afraid, preclude publication of the manuscript in EMBO Molecular Medicine. I will not discuss each point in detail as they are clearly stated.

Briefly, Reviewer 1's main concern is the lack of insight into the mechanism of action of 5-azacytidine. While detailed mechanistic analysis is not per se a precondition for publication in EMBO Molecular Medicine (provided of course that translational value is demonstrated), in this case the question if 5-azacytidine acts directly or indirectly on NMD does remain completely open. Reviewer 1 also notes a potential experimental flaw due to the use of an apparently intron-less reporter construct, which would not recruit NMD factors.

Reviewer 2 is in general concerned about the limited medical impact of the current dataset. As mentioned above, while extensive mechanistic insight is not an absolute requirement when the clinical implications and conceptual advance are striking, we agree with this Reviewer that the latter criteria are not met and thus additional, more clinically relevant data would be required. This might include, for example, testing and validating 5-azacytidine on appropriate cell models of PTC-linked disease and verifying NMD in blood samples from 5-azacytidine-treated leukaemia patients. Such experimental support would considerably increase the significance and impact of your work.

Reviewer 2 lists other important concerns related to experimental controls and procedures, and the validation of the drugs identified.

The lack of the mechanistic insight is clearly a leitmotif here and indeed Reviewer 3 also feels that the lack of any positive mechanistic data renders the manuscript incomplete at this stage. Concerning the clinical implications, Reviewer 3 further notes the lack of information as to why/if 5-azacytidine is better than any of the drugs currently in use. S/he also challenges some of the experimental approaches: a case in point is the alleged questionable efficacy of PTC124, which is felt, similarly to Reviewer 2, to be inappropriate. Finally, Reviewer 3 also lists other issues related to experimental controls and procedures.

Given these fundamental concerns and the overall lack of enthusiasm by the reviewers, we feel we have no choice but to return the manuscript to you at this stage. In our assessment it is not realistic to expect to be able to address these issues experimentally in a reasonable time frame and to the satisfaction of the Reviewers.

I am sorry to have to disappoint you at this stage. I hope that the Reviewers' comments will be helpful in your continued work in this area.
***** Reviewer's comments *****

Referee #1 (Comments on Novelty/Model System):

Well-performed experiments addressing a novel and potentially very important finding. Medical impact is medium (see comments to authors) and model system is adequate although animal models will be needed at later stages.

Referee #1 (Remarks):

The manuscript by Bhuvanagiri et al describes the interesting and surprising observation that 5-azacytidine, a drug normally used for targeting of aberrant DNA methylation in leukemia and MDS, inhibits the nonsense-mediated decay (NMD) pathway.
The authors used a reporter based chemical screen to identify 5-azacytidine as the single drug (among >1.000) capable of selectively stabilizing a NMD sensitive mRNA. Interestingly, this property was unique to 5-azacytidine among a collection of 20 structurally similar compounds and 5-azacytidine was further shown to inhibit NMD in the micromolar range. Next, the findings from the NMD sensitive reporter system were extended to a number of previously characterized endogenous NMD target, demonstrating that 5-azacytidine seem to broadly inhibit NMD.
The authors then seek to address the underlying mechanisms by which 5-azacytidine inhibit the NMD pathway. First, they demonstrate that 5-azacytidine had no effect on the expression of a number of well-established NMD components. Similarly 5-azacytidine did not affect the phosphorylation of UPF1, a key event during activation of the NMD pathway. Secondly, they show that 5-azacytidine did not affect translation per se, which is important since the NMD pathway is dependent on active translation. Thirdly, they demonstrate that 5-azacytidine does not promote translational readthrough. Finally, they show that 5-azacytidine has not effect on the expression of the reporter pre-mRNA. This is a particularly important experiment as 5-azacytidine affects the methylation status of DNA, which may lead to downregulation of pre-mRNA upstream i.e. of the NMD pathway.
Collectively the work by Bhuvanagiri is well controlled and describe a very interesting finding, however a number of major concerns will have to be addressed.

Major points:
1. A clear weakness of the manuscript in its present form is the lack of mechanism. The authors have tested the most common mechanisms by which NMD can be inhibited and should be credited for these efforts. However, without any clue of the mechanism the manuscript is having, in my opinion, a hard time meeting the high standards of EMBO Molecular Medicine. As a minimum the authors should try to combine knockdown of key NMD components acting at distinct steps during the NMD pathway with and without 5-azacytidine treatments. This should allow them to determine potential synergies between inhibition of distinct NMD components and 5-azacytidine and provide insights into the steps at which 5-azacytidine is acting.

Along the same lines, the authors forward the hypothesis that 5-azacytidine may interfere with methylation status of RNA. It is not known to this reviewer to which extent cytidines in RNA are modified, but if so this is an extremely concept, which could be tested both by assessing the methylation status of RNA and/or by overexpression/knockdown experiments of enzymes involved in the generation/removal of methyl groups in RNA.

2. The second major concern is more of conceptual nature. The authors argue that 5-azacytidine may be an interesting drug for treatment of various diseases but how do they reconcile such a strategy with the extremely harsh KO phenotypes of NMD deficient mice (such as UPF1, UPF2, SMG1). The authors should discuss this and also cite the mouse work as this provides important clues for the tolerance of strong NMD inhibition.

3. I´m a concerned that the conclusions with respect to the effect of 5-azacytidine on translational read-through is drawn from what appear to be an intronless reporter construct, which will not recruit any NMD factors. Consequently, such a reporter cannot be used to assess readthrough of a NMD-inducing stop-codon. The authors should either re-design this reporter, or alternatively, perform western blot analysis on lysates expressing the reporter in Fig 1.A.

Minor points:
1. Abstract: The sentence starting with "Here, we uncover..." is unclear. Please rephrase.

2. Fig 3 panel A. Please align the numbers below the gel properly.

Referee #2 (Comments on Novelty/Model System):

Technical quality is rated as medium because the authors have not addressed the possible inhibition of protein synthesis sufficiently, have not explained their initial assay in adequate detail, and have not indicated whether the identity of the 5-azacytidine obtained from the commercial library was confirmed.

Novelty is listed as medium because the Lejeune and Bedwell groups have already published papers suggesting that NMD inhibition had therapeutic potential.

Medical impact is listed as low because extensive clinical testing must precede implementation of the information presented here.

Referee #2 (Remarks):

Bhuvanagiri et al describe experiments intended to identify new inhibitors of nonsense-mediated mRNA decay (NMD). They justify their search by noting that such inhibitors have the potential to serve as disease-modifying agents for those disorders attributable to premature termination codons (PTCs). A commercial library of 1120 compounds that have undergone some degree of clinical testing was screened and 5-azacytidine emerged as a possible NMD inhibitor. This is potentially an interesting result, but the manuscript brought to light several issues that must be addressed before publication can be considered. These issues include:

1. In Figure 1 the authors present data intended to establish the credibility of their assay system. Cultured cells were treated with known inhibitors of NMD, included the protein synthesis inhibitors anisomycin and cycloheximide. In subsequent steps the authors then assayed luciferase activity from their reporters, showing that the PTC-containing reporter yielded enhanced luciferase activity upon treatment with both inhibitors. While it's understandable that the reporter mRNA levels may have increased it is not at all clear from the details provided for the assay how enhanced LUC activity could have been expressed in the presence of inhibitors of protein synthesis. Some details must be missing and the authors need to provide them.

2. Although the fold-increase data provided in Figure 1 are interesting, the relevant value for any drug of clinical potential is the extent of restoration of wild-type levels. Hence, the data of Figure 1D and F (as well as several other figures) needs to include wild-type controls so that the reader can assess the percentage of wild-type activity resulting from drug treatment.

3. The question of possible protein synthesis inhibition is of critical importance because of the possibility that 5-azacytidine acts indirectly. Hence, the approach used in Figure 5 is necessary, but, as presented, it is not sufficient. The authors have addressed a critical variable with only single-concentration assays. As reflected by the modest inhibition of cycloheximide (a potent inhibitor of protein synthesis) in Figure 5A, these single point assays are inadequate. They should be replaced by analyses of a broad range of doses, using both the assays presented and an additional assay, namely polyribosome profiles. The latter are particularly sensitive to inhibition of protein synthesis and can even indicate whether inhibition (if it occurs) affects initiation or elongation. It is highly recommended that cellular growth rates with and without 5-azaC also be evaluated.

4. In light of the authors' introductory and concluding remarks implying that modulation of NMD has therapeutic potential there must also be a strong cautionary note that little is presently understood about the consequences of modulating the levels of mRNAs normally degraded by NMD. It should be pointed out that since NMD is a surveillance pathway there is a possibility that inactivating its function may be toxic in patients subjected to treatment regimens differing from those currently approved for cancer therapy.

5. While we all tend to trust our suppliers of chemicals it is customary in drug screening to ascertain that a compound identified in a library is indeed the chemical entity it is thought to be. Hence, have the authors confirmed that the compound from the library is indeed 5-azacytidine? Stated another way: have the authors used independent sources of the compound?

6. The Introduction could use a little updating. The references to review articles on NMD in the first sentence and the later comment suggesting that the efficacy of PTC124 is questionable are both out of date. There are many newer review articles on NMD and, in 2013 alone, there have been at least five papers demonstrating readthrough activity of PTC124 with multiple mRNAs in diverse biological systems.

7. Trivia: cycloheximide is misspelled several times.

Referee #3 (Comments on Novelty/Model System):

The study is technically well done. This would fit best in a Novelty category between medium and high. It is novel in that it identified an existing drug as a potent inhibitor of NMD, but this is mitigated by the fact that there are other drugs that are also effective that are in clinical use. This is also why I rated medical impact as medium, since it is not clear that 5-azacytidine is sufficiently better. I also felt the Introduction was disingenuous in the manner in which the authors framed the current state of drugs that are being evaluated as inhibitors of NMD. The model system is fine.

Referee #3 (Remarks):

The manuscript by Bhuvanagiri et al. describes a screen for inhibitors of NMD using HeLa cells stably expressing Renilla luciferase fused to a human beta-globin gene with a premature termination codon at position 39. The authors identified 6 candidates from a screen of 1120 compounds, the most active of which was 5-azacytidine. Given that this is already in clinical use, its identification raises the possibility of a new approach for therapeutic intervention in diseases caused by nonsense codons. They show that 5-azacytidine selectively increases the steady-state level of nonsense-containing beta-globin mRNA without altering the level of pre-mRNA, that it upregulates the levels of endogenous NMD targets, and propose that its mechanism of action is independent of changes in the levels of a number of NMD or EJC components, the phosphorylation state of Upf1, the overall translation state of the cell, or translational read-through. However, the actual mechanism by which 5-azacytidine inhibits NMD is not presented.

Major comments:

1) Page 5, paragraph 1: the authors cite a paper questioning the efficacy of PTC124 (Auld et al, 2009) as a reason for identifying new inhibitors of NMD, and cast this in the context of inhibitors of other NMD factors that are 'too toxic for clinical use or proved to be ineffective.' Results in Auld et

al. have been disputed, and since that time a number of publications have appeared that support the efficacy of PTC124 (eg. Goldmann, T. et al. EMBO Mol. Med. 4, 1186-1199, 2012). The text should be revised to provide a more evenhanded representation of the state of the field.

2) Experiments in Figure 3 used an 18 hr treatment with 5-azacytidine to show that it acts at the post-transcriptional level to increase the amount of nonsense-containing beta-globin mRNA without affecting the amount of pre-mRNA. This seems to be a rather prolonged treatment. What happens when cells are exposed for a shorter period of time? Also, the results would be strengthened by experiments showing the impact of 5-azacytidine on decay rate.

3) A major premise of this study is that 5-azacytidine inhibits NMD through a novel mechanism that is independent of NMD factors. However, Upf3A is clearly increased (Fig. 4A) and there is nothing in the manuscript about SMG6 even though this is a major catalytic effector of NMD.

4) Although Figs. 5 and 6 provide convincing evidence that 5-azacytidine does not have a generalized effect on translation or on translational read-through, these are essentially negative results. The study is incomplete without the actual mechanism by which 5'-azacytidine inhibits NMD.

Minor comments;

5) In Fig. 1D it is surprising that Wortmanin had no effect on their NMD reporter, particularly since its effect on Upf1 phosphorylation is evident in Fig. 4C. Since Wortmanin has been used in numerous studies to inhibit NMD the authors should provide an explanation for the absence of its effect in their system.

6) Page 8, paragraph 1: The reference to Figure 2A for experiments with serial dilutions of nucleoside analogs is confusing. That figure is a schematic showing beta-globin gene constructs.

Resubmission 22 July 2014

Referee #1 (Comments on Novelty/Model System):

Well-performed experiments addressing a novel and potentially very important finding. Medical impact is medium (see comments to authors) and model system is adequate although animal models will be needed at later stages. Referee #1 (Remarks): The manuscript by Bhuvanagiri et al describes the interesting and surprising observation that 5-azacytidine, a drug normally used for targeting of aberrant DNA methylation in leukemia and MDS, inhibits the nonsense-mediated decay (NMD) pathway.
The authors used a reporter based chemical screen to identify 5-azacytidine as the single drug (among >1.000) capable of selectively stabilizing a NMD sensitive mRNA. Interestingly, this property was unique to 5-azacytidine among a collection of 20 structurally similar compounds and 5-azacytidine was further shown to inhibit NMD in the micromolar range. Next, the findings from the NMD sensitive reporter system were extended to a number of previously characterized endogenous NMD target, demonstrating that 5-azacytidine seem to broadly inhibit NMD.
The authors then seek to address the underlying mechanisms by which 5-azacytidine inhibit the NMD pathway. First, they demonstrate that 5-azacytidine had no effect on the expression of a number of well-established NMD components. Similarly 5-azacytidine did not affect the phosphorylation of UPF1, a key event during activation of the NMD pathway. Secondly, they show that 5-azacytidine did not affect translation per se, which is important since the NMD pathway is dependent on active translation. Thirdly, they demonstrate that 5-azacytidine does not promote translational readthrough. Finally, they show that 5-azacytidine has not effect on the expression of the reporter pre-mRNA. This is a particularly important experiment as 5-azacytidine affects the methylation status of DNA, which may lead to downregulation of pre-mRNA upstream i.e. of the NMD pathway.
Collectively the work by Bhuvanagiri is well controlled and describe a very interesting finding, however a number of major concerns will have to be addressed.

Major points:

1. A clear weakness of the manuscript in its present form is the lack of mechanism. The authors have tested the most common mechanisms by which NMD can be inhibited and should be credited for these efforts. However, without any clue of the mechanism the manuscript is having, in my opinion, a hard time meeting the high standards of EMBO Molecular Medicine. As a minimum the authors should try to combine knockdown of key NMD components acting at distinct steps during the NMD pathway with and without 5-azacytidine treatments. This should allow them to determine potential synergies between inhibition of distinct NMD components and 5-azacytidine and provide insights into the steps at which 5-azacytidine is acting.

We have now addressed the mechanism by which 5-azacytidine inhibits NMD. We performed a global mass spectrometric analysis and identified proteins that are up/down regulated upon 5-azacytidine treatment when compared to negative controls including the chemically very closely related 5-aza-2' deoxycytidine. Our analysis revealed 21 proteins to be significantly upregulated and 32 proteins to be significantly downregulated upon 5-azacytidine treatment, as explained in the new Figure 7C and D. Importantly, by RNAi experiments (shown in the new Figure 8) we uncover the importance of MYC activation for NMD inhibition by 5-azacytidine.

Along the same lines, the authors forward the hypothesis that 5-azacytidine may interfere with methylation status of RNA. It is not known to this reviewer to which extent cytidines in RNA are modified, but if so this is an extremely concept, which could be tested both by assessing the methylation status of RNA and/or by overexpression/knockdown experiments of enzymes involved in the generation/removal of methyl groups in RNA.

We appreciate this interesting suggestion and have thus performed overexpression and RNAi experiments depleting the two most studied RNA methyltransferases, TRDMT1 and NSUN2. However, our results did not show any specific upregulation of NMD targets. Hence, we have decided not to include these essentially negative data in this manuscript.

2. The second major concern is more of conceptual nature. The authors argue that 5-azacytidine may be an interesting drug for treatment of various diseases but how do they reconcile such a strategy with the extremely harsh KO phenotypes of NMD deficient mice (such as UPF1, UPF2, SMG1). The authors should discuss this and also cite the mouse work as this provides important clues for the tolerance of strong NMD inhibition.

We totally agree with the reviewer that a complete inactivation of the NMD factors UPF1 and UPF2 yields embryonically lethal phenotypes in mice. However, the intention of the study is to identify compounds that modulate but not completely inactivate NMD activity.

3.I´m a concerned that the conclusions with respect to the effect of 5-azacytidine on translational read-through is drawn from what appear to be an intronless reporter construct, which will not recruit any NMD factors. Consequently, such a reporter cannot be used to assess readthrough of a NMD-inducing stopcodon. The authors should either re-design this reporter, or alternatively, perform western blot analysis on lysates expressing the reporter in Fig 1.A.

We appreciate it that an intronless reporter does not recruit proteins that are deposited on the RNA by the splicing process (such as exon junction complex proteins). However, we used the intronless reporter to assess the effect of 5-azacytidine on readthrough together with appropriate negative and positive controls. These in our opinion are well controlled experiments that show 5-azacytidine does not augment readthrough, and we thus propose to keep these data in the manuscript.

Minor points:
1. Abstract: The sentence starting with "Here, we uncover..." is unclear. Please rephrase.
As suggested, we have rephrased this sentence.

2. Fig 3 panel A. Please align the numbers below the gel properly.
As suggested, we have now re-aligned the numbers below the gel

Referee #2 (Comments on Novelty/Model System):

Technical quality is rated as medium because the authors have not addressed the possible inhibition of protein synthesis sufficiently, have not explained their initial assay in adequate detail, and have not indicated whether the identity of the 5-azacytidine obtained from the commercial library was confirmed.

Novelty is listed as medium because the Lejeune and Bedwell groups have already published papers suggesting that NMD inhibition had therapeutic potential.

Medical impact is listed as low because extensive clinical testing must precede implementation of the information presented here.

Referee #2 (Remarks):

Bhuvanagiri et al describe experiments intended to identify new inhibitors of nonsense-mediated mRNA decay (NMD). They justify their search by noting that such inhibitors have the potential to serve as disease-modifying agents for those disorders attributable to premature termination codons (PTCs). A commercial library of 1120 compounds that have undergone some degree of clinical testing was screened and 5-azacytidine emerged as a possible NMD inhibitor. This is potentially an interesting result, but the manuscript brought to light several issues that must be addressed before publication can be considered. These issues include:

1. In Figure 1 the authors present data intended to establish the credibility of their assay system. Cultured cells were treated with known inhibitors of NMD, included the protein synthesis inhibitors anisomycin and cycloheximide. In subsequent steps the authors then assayed luciferase activity from their reporters, showing that the PTC-containing reporter yielded enhanced luciferase activity upon treatment with both inhibitors. While it's understandable that the reporter mRNA levels may have increased it is not at all clear from the details provided for the assay how enhanced LUC activity could have been expressed in the presence of inhibitors of protein synthesis. Some details must be missing and the authors need to provide them.

We are grateful for this comment. In response, we have now added new data and show in the new Supplementary Figure 1 results of a dose-response analysis of the translation inhibitor anisomycin that was used as a positive control. We have carefully determined the concentrations of anisomycin, at which there is maximum NMD inhibition and minimal translation inhibition of our luciferase based studies. To further confirm the effect of anisomycin at selected concentrations on the stability of RNA while translation remains active, we have also performed Northern blot analysis and our results show a very strong upregulation of PTC-burdened mRNAs at the selected concentrations.

2. Although the fold-increase data provided in Figure 1 are interesting, the relevant value for any drug of clinical potential is the extent of restoration of wild-type levels. Hence, the data of Figure 1D and F (as well as several other figures) needs to include wild-type controls so that the reader can assess the percentage of wild-type activity resulting from drug treatment.

We appreciate this important comment. However, particularly for the Figures 1D and 1F, we chose to represent only NMD mutant data because the ratio of mutant to wildtype was misleading for compounds such as emetine and cephealine, which are known translation inhibitors. Normalization to the wildtype thus led to a high false positive ratio. We have thus opted for maintaining the original presentation of the data in panels D and F of Figure 1. However, we have included a new panel A in Supplementary Figure 3 showing that 5-azacytidine results in the synthesis of approx. 40% of the wildtype protein at a concentration of 1.56 uM.

3. The question of possible protein synthesis inhibition is of critical importance because of the possibility that 5-azacytidine acts indirectly. Hence, the approach used in Figure 5 is necessary, but, as presented, it is not sufficient. The authors have addressed a critical variable with only single-concentration assays. As reflected by the modest inhibition of cycloheximide (a potent inhibitor of protein synthesis) in Figure 5A, these single point assays are inadequate. They should be replaced by analyses of a broad range of doses, using both the assays presented and an additional assay, namely polyribosome profiles. The latter are particularly sensitive to inhibition of protein synthesis

and can even indicate whether inhibition (if it occurs) affects initiation or elongation. It is highly recommended that cellular growth rates with and without 5-azaC also be evaluated.

We agree with this reviewer that inhibition of protein synthesis by 5-azacytidine must be rigorously excluded as a potential mechanism of its action. We have therefore performed dose response experiments with the wildtype reporter showing that, expectedly, high concentrations of 5-azacytidine indeed inhibit translation (Figure 2A). However, we have performed the detailed analyses of translation inhibition at concentrations at which we confirmed the maximal effect of 5-azacytidine on NMD efficiency and minimal toxicity. Similarly, we chose concentrations of anisomycin and cycloheximide which show NMD inhibition but only minor translation inhibition. As suggested, we have now assessed a potential inhibition of translation by 5-azacytidine via polysomal analysis. These new data are now shown in the new panel D of Figure 5. This analysis further confirms that at low concentrations 5-azacytidine does not inhibit translation.

4. In light of the authors' introductory and concluding remarks implying that modulation of NMD has therapeutic potential there must also be a strong cautionary note that little is presently understood about the consequences of modulating the levels of mRNAs normally degraded by NMD. It should be pointed out that since NMD is a surveillance pathway there is a possibility that inactivating its function may be toxic in patients subjected to treatment regimens differing from those currently approved for cancer therapy.

As suggested, we have now explained the scope of potential clinical use of 5-azacytidine more explicitly in the discussion section.

5. While we all tend to trust our suppliers of chemicals it is customary in drug screening to ascertain that a compound identified in a library is indeed the chemical entity it is thought to be. Hence, have the authors confirmed that the compound from the library is indeed 5-azacytidine? Stated another way: have the authors used independent sources of the compound?

We agree with the reviewer that the authenticity of the compounds used needs to be evaluated. 5-azacytidine is known to down regulate DNA methyl transferase protein (DNMT1). We have thus confirmed this activity by Western blot analysis of DNMT1 with every batch of 5-azacytidine we used. We have now mentioned this in the methods section.

6. The Introduction could use a little updating. The references to review articles on NMD in the first sentence and the later comment suggesting that the efficacy of PTC124 is questionable are both out of date. There are many newer review articles on NMD and, in 2013 alone, there have been at least five papers demonstrating readthrough activity of PTC124 with multiple mRNAs in diverse biological systems.

As suggested we have changed the introduction and updated our references.

7. Trivia: cycloheximide is misspelled several times.

We have now corrected the spelling of cycloheximide

Referee #3 (Comments on Novelty/Model System):

The study is technically well done. This would fit best in a Novelty category between medium and high. It is novel in that it identified an existing drug as a potent inhibitor of NMD, but this is mitigated by the fact that there are other drugs that are also effective that are in clinical use. This is also why I rated medical impact as medium, since it is not clear that 5-azacytidine is sufficiently better. I also felt the Introduction was disingenuous in the manner in which the authors framed the current state of drugs that are being evaluated as inhibitors of NMD. The model system is fine.

Referee #3 (Remarks):

The manuscript by Bhuvanagiri et al. describes a screen for inhibitors of NMD using HeLa cells

stably expressing Renilla luciferase fused to a human beta-globin gene with a premature termination codon at position 39. The authors identified 6 candidates from a screen of 1120 compounds, the most active of which was 5-azacytidine. Given that this is already in clinical use, its identification raises the possibility of a new approach for therapeutic intervention in diseases caused by nonsense codons. They show that 5-azacytidine selectively increases the steady-state level of nonsense-containing beta-globin mRNA without altering the level of pre-mRNA, that it upregulates the levels of endogenous NMD targets, and propose that its mechanism of action is independent of changes in the levels of a number of NMD or EJC components, the phosphorylation state of Upf1, the overall translation state of the cell, or translational read-through. However, the actual mechanism by which 5-azacytidine inhibits NMD is not presented.

Major comments:

1) Page 5, paragraph 1: the authors cite a paper questioning the efficacy of PTC124 (Auld et al, 2009) as a reason for identifying new inhibitors of NMD, and cast this in the context of inhibitors of other NMD factors that are 'too toxic for clinical use or proved to be ineffective.' Results in Auld et al. have been disputed, and since that time a number of publications have appeared that support the efficacy of PTC124 (eg. Goldmann, T. et al. EMBO Mol. Med. 4, 1186-1199, 2012). The text should be revised to provide a more evenhanded representation of the state of the field.

As suggested by the reviewer, the text has been revised and updated.

2) Experiments in Figure 3 used an 18 hr treatment with 5-azacytidine to show that it acts at the post-transcriptional level to increase the amount of nonsense-containing beta-globin mRNA without affecting the amount of pre-mRNA. This seems to be a rather prolonged treatment. What happens when cells are exposed for a shorter period of time? Also, the results would be strengthened by experiments showing the impact of 5-azacytidine on decay rate.

Preliminary time course and dose response experiments revealed that 5-azacytidine showed its maximum activity and minimal toxicity at 1.56μM concentration and 18 hours. Hence, 18 hour time point was used all through the study

3) A major premise of this study is that 5-azacytidine inhibits NMD through a novel mechanism that is independent of NMD factors. However, Upf3A is clearly increased (Fig. 4A) and there is nothing in the manuscript about SMG6 even though this is a major catalytic effector of NMD.

We are grateful to this reviewer for pointing this out. The original panel of Figure 4A suffered from unequal sample loading. We have now repeated these analyses multiple times and replaced the old panel by a new version of better technical quality. It confirms and shows more clearly that 5-azacytidine does not affect the abundance of UPF3A. Regarding SMG6, commercially available antibodies that we tested failed to yield quantifiable results for SMG6. However, the proteomic analyses (shown in the Figure. 7) showed no change of SMG6 and also of the other NMD proteins.

4) Although Figs. 5 and 6 provide convincing evidence that 5-azacytidine does not have a generalized effect on translation or on translational read-through, these are essentially negative results. The study is incomplete without the actual mechanism by which 5'-azacytidine inhibits NMD.

Our new data (Figure 7 and 8) now show that MYC is required for the effect of 5-azacytidine on NMD.

Minor comments:

5) In Fig. 1D it is surprising that Wortmannin had no effect on their NMD reporter, particularly since its effect on Upf1 phosphorylation is evident in Fig. 4C. Since Wortmannin has been used in numerous studies to inhibit NMD the authors should provide an explanation for the absence of its effect in their system.

We found the inhibition of NMD by wortmannin to be variable. We have therefore used cycloheximide and anisomycin as more robust positive controls for our studies. In order to avoid

ambiguity, we have now removed wortmannin from Figure 1D.

6) Page 8, paragraph 1: The reference to Figure 2A for experiments with serial dilutions of nucleoside analogs is confusing. That figure is a schematic showing beta-globin gene constructs.

As suggested by the reviewer, Figure 2A has now been changed.

2nd Editorial Decision 22 August 2014

Thank you for the re-submission of your revised manuscript to EMBO Molecular Medicine. We have now received the enclosed reports from the reviewers that were asked to re-assess it. As you will see, while two are now supportive, Reviewer 2 still feels that the mechanistic insight provided is insufficient. However, after discussion with my colleagues, and considering the translational interest of your findings, I am pleased to inform you that we will be able to accept your manuscript.

Please submit your revised manuscript within two weeks. I look forward to seeing a revised form of your manuscript as soon as possible.

***** Reviewer's comments *****

Referee #1 (Comments on Novelty/Model System):

Beautifully done study with high potential medical impact, given that 5AzaC is already FDA approved and the authors show it is a potent NMD inhibitor. Novelty is medium to high; only reason it is not "high" is because other NMD inhibitors have been identified.

Referee #1 (Remarks):

The authors have done an excellent job of responding the Reviewers' comments. Most importantly, their revised manuscript reveal a mechanism for 5AzaC action. The authors demonstrate that 5AzaC acts by upregulating the expression of the well known oncogene c-myc.

Referee #2 (Remarks):

My main concern in the first version of the manuscript by xxx was the lack of mechanistic explanation for why 5-azacytidine inhibits NMD. In the revised version the authors now show that 5-aza leads to the up-regulation of MYC and that down-regulation of MYC abrogate the effect of 5-aza on NMD. Whereas this finding confirms earlier findings demonstrating that MYC inhibits NMD it does not explain how 5-aza triggers increased expression of MYC nor does it explain how MYC selectively stabilize PTC containing transcripts. In my opinion the lack of mechanistic insights into the action of 5´aza prohibit publication in EMBO Molecular Medicine.

Referee #3 (Comments on Novelty/Model System):

see previous review

Referee #3 (Remarks):

The authors have addressed previous criticisms.
