# Supplementary figures and images for: 5-azacytidine inhibits nonsense-mediated decay in a MYC-dependent fashion
Source: EMBO Mol Med. 2014 Oct 15;6(12):1593–609. doi: 10.15252/emmm.201404461 (PMC4287977; doi:10.15252/emmm.201404461)

Figure.4A

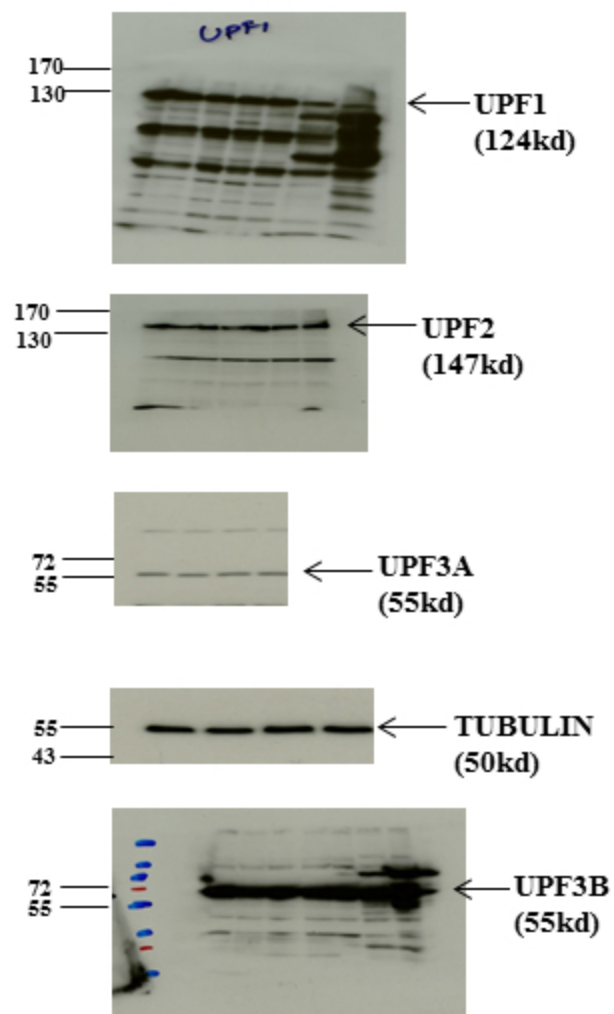

Figure.4B

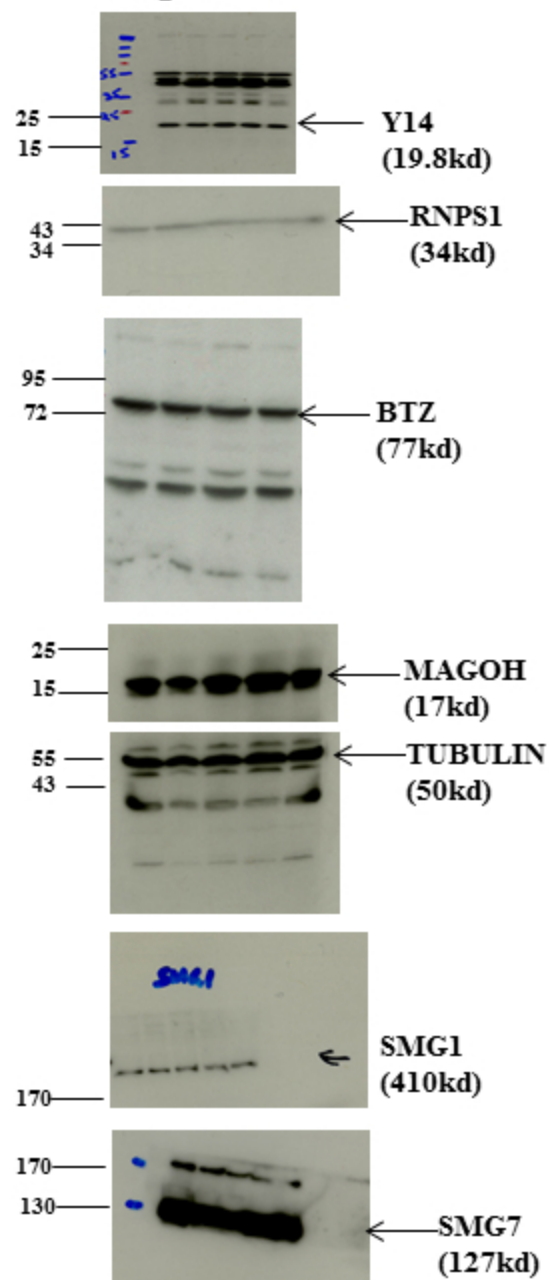

Figure.4C

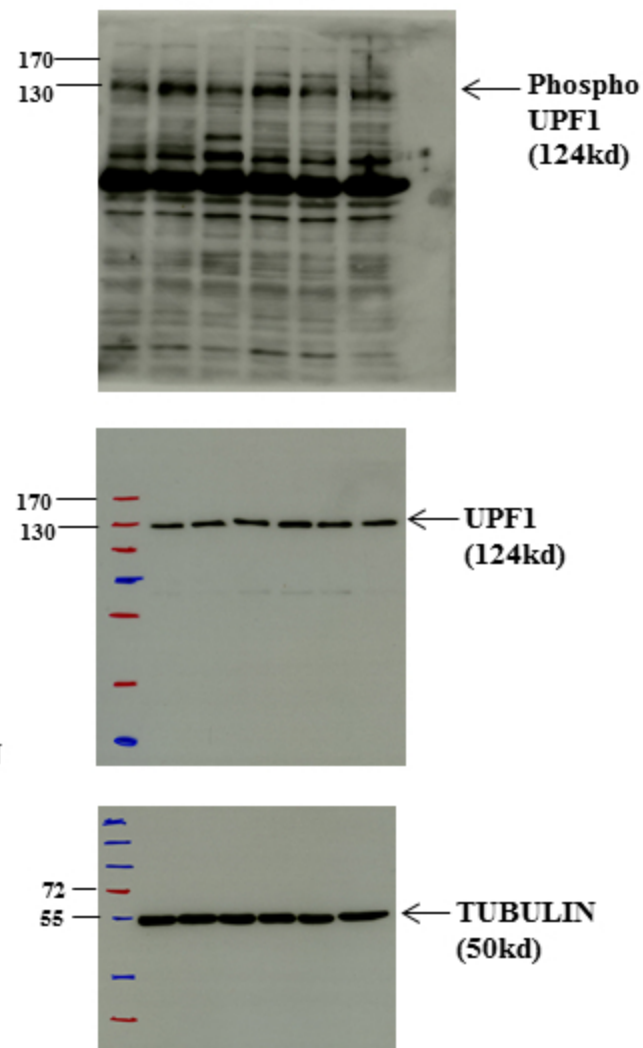

Supplement: Supplementary file 3 — Source Data for Figure 1 B [file emmm0006-1593-sd3.pdf]

Figure.5 B and C

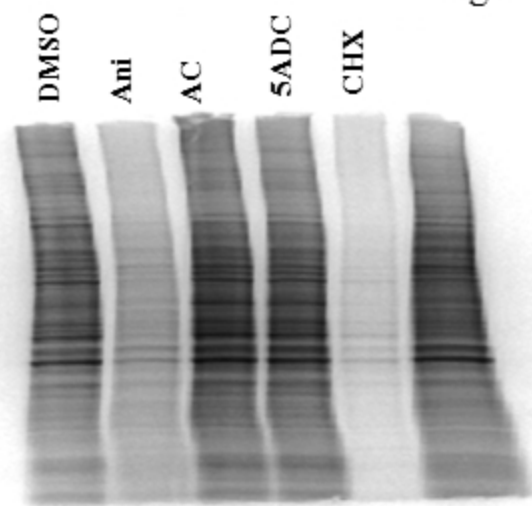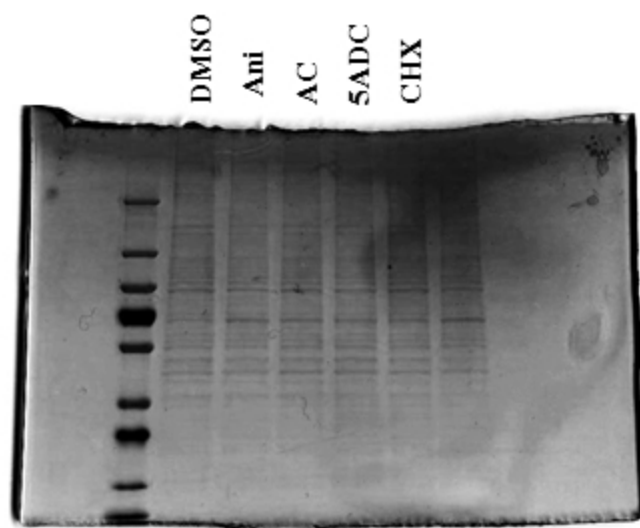

Supplement: Supplementary file 4 — Source Data for Figure 4 A B C [file emmm0006-1593-sd4.pdf]

Figure.8B

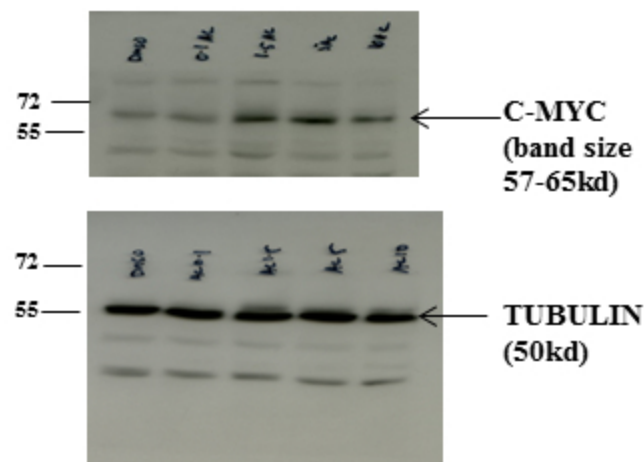

Figure.8F

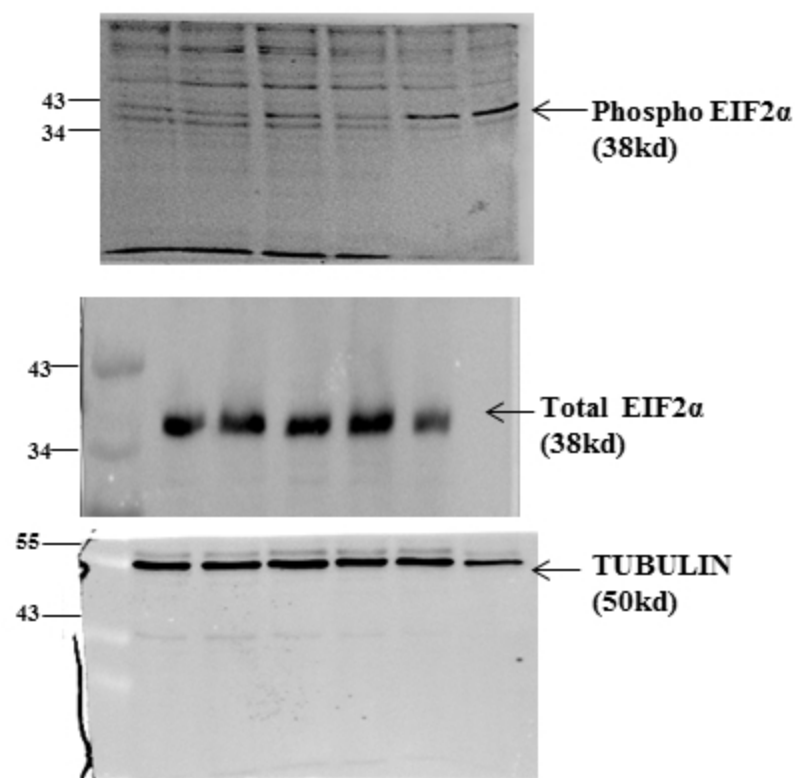

Supplement: Supplementary file 5 — Source Data for Figure 5 B C [file emmm0006-1593-sd5.pdf]
