# Supplementary material for: 5-azacytidine inhibits nonsense-mediated decay in a MYC-dependent fashion
Source: EMBO Mol Med. 2014 Oct 15;6(12):1593–609. doi: 10.15252/emmm.201404461 (PMC4287977; doi:10.15252/emmm.201404461)
Supplement: Supplementary file 6 — Source Data for Figure 8 B F [file emmm0006-1593-sd6.pdf]

Supplementary Figures

Fig.s1

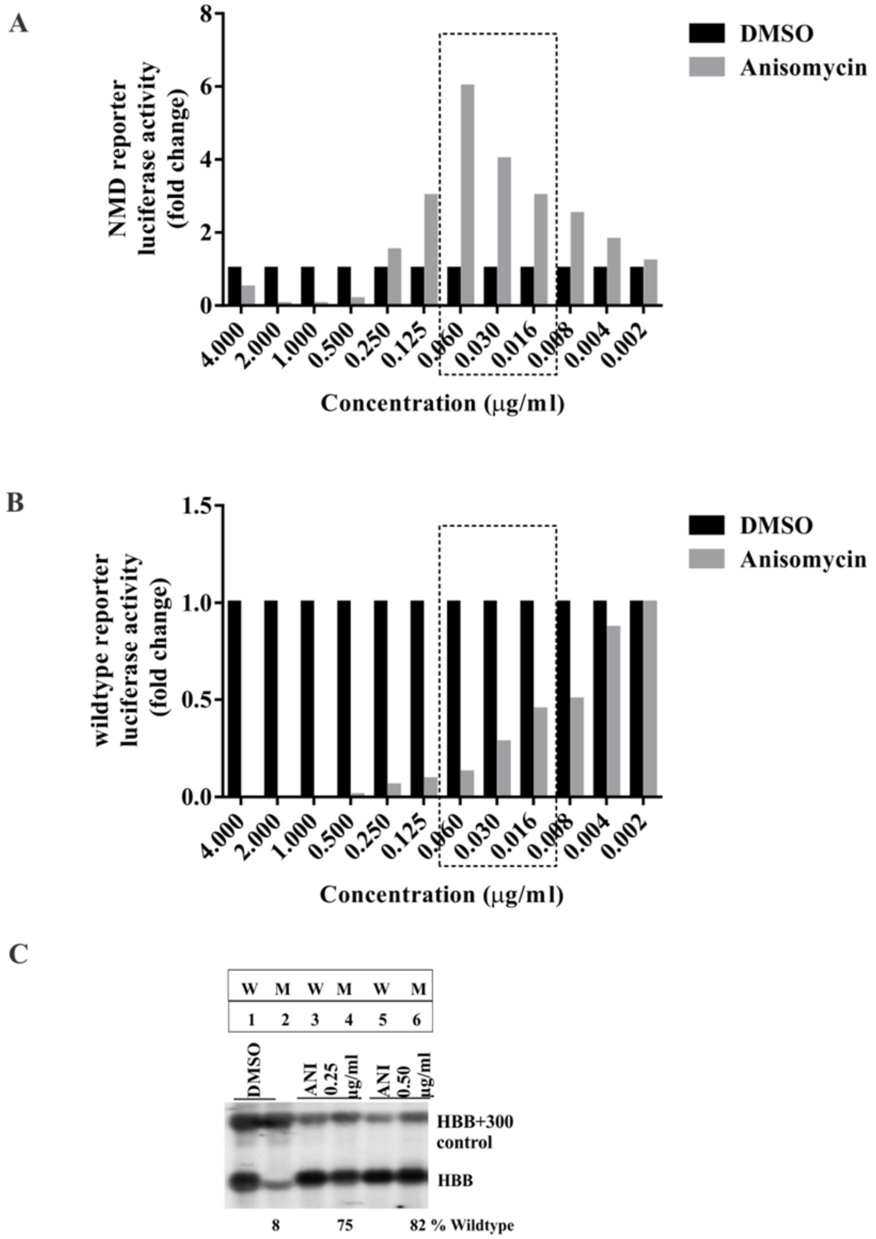

Supplementary Figure S1: Dose response studies with anisomycin reveals that maximum NMD inhibition occurs at low concentrations that only moderately inhibit translation.

(**A**) A dose response study using anisomycin was performed on Hela cells stably expressing PTC-mutated HBB gene fused to renilla luciferase reporter. The bar diagram shows that at higher concentrations there is no upregulation of the NMD reporter, because of the global inhibition of translation. However, at lower concentrations a dose dependent upregulation of the NMD reporter is noticed. The dotted box shows the concentrations of anisomycin at which the maximum inhibition of NMD is observed.

(**B**) Hela cells stably expressing a wildtype HBB gene fused to renilla luciferase were tested to select the appropriate concentration of anisomycin at which there is maximum inhibition of NMD and sufficient remaining activity of translation to enable expression of the luciferase reporter (dotted box).

(**C**) Northern blot of total cellular RNA of HeLa cells stably expressing wildtype (W) or PTC-mutated (M) HBB genes following treatment with DMSO, 0.25 µg/ml anisomycin (ANI) or 0.50 µg/ml anisomycin for 18 hours. HBB+300 was co-transfected as a loading control. The expression of PTC-mutated HBB reporter mRNA is shown in % of wildtype.

Fig.s2

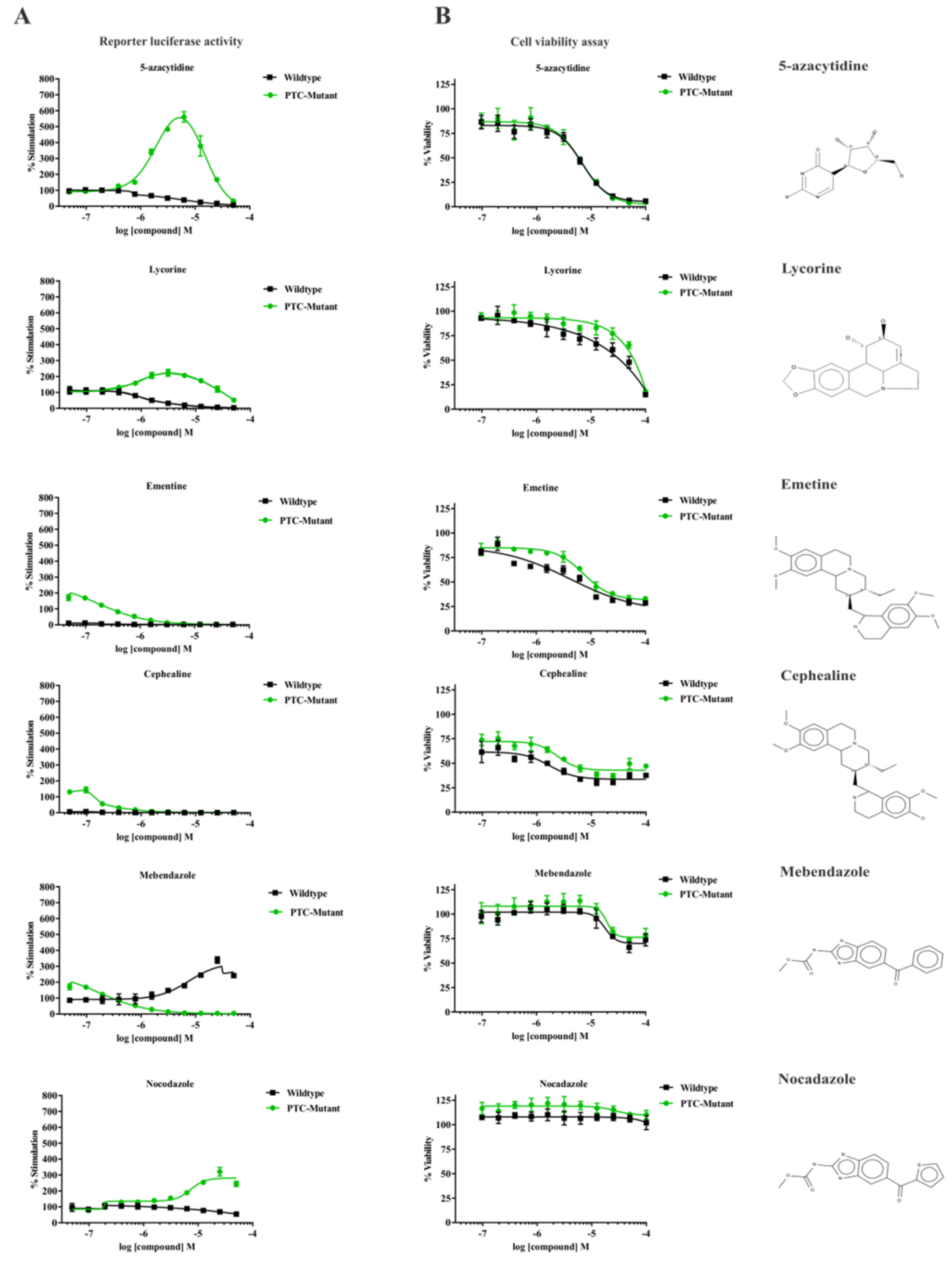

Supplementary Figure S2: Dose response and cytotoxicity assay of the selected hits tested on HeLa cells expressing either wildtype or PTC-mutated reporters.

(**A**) The graphs on the left represent the luciferase activity of HBB wildtype (black) or PTC-mutant (green) fusion reporters. The X–axis represents the serial dilution of the compounds and the Y-axis shows percentage (%) stimulation of luciferase activity. (**B**) The graphs on the right represent viability of HeLa cells expressing the wildtype or mutant HBB reporters. The X–axis represents the serial dilution of the compounds and the Y-axis shows % viability.

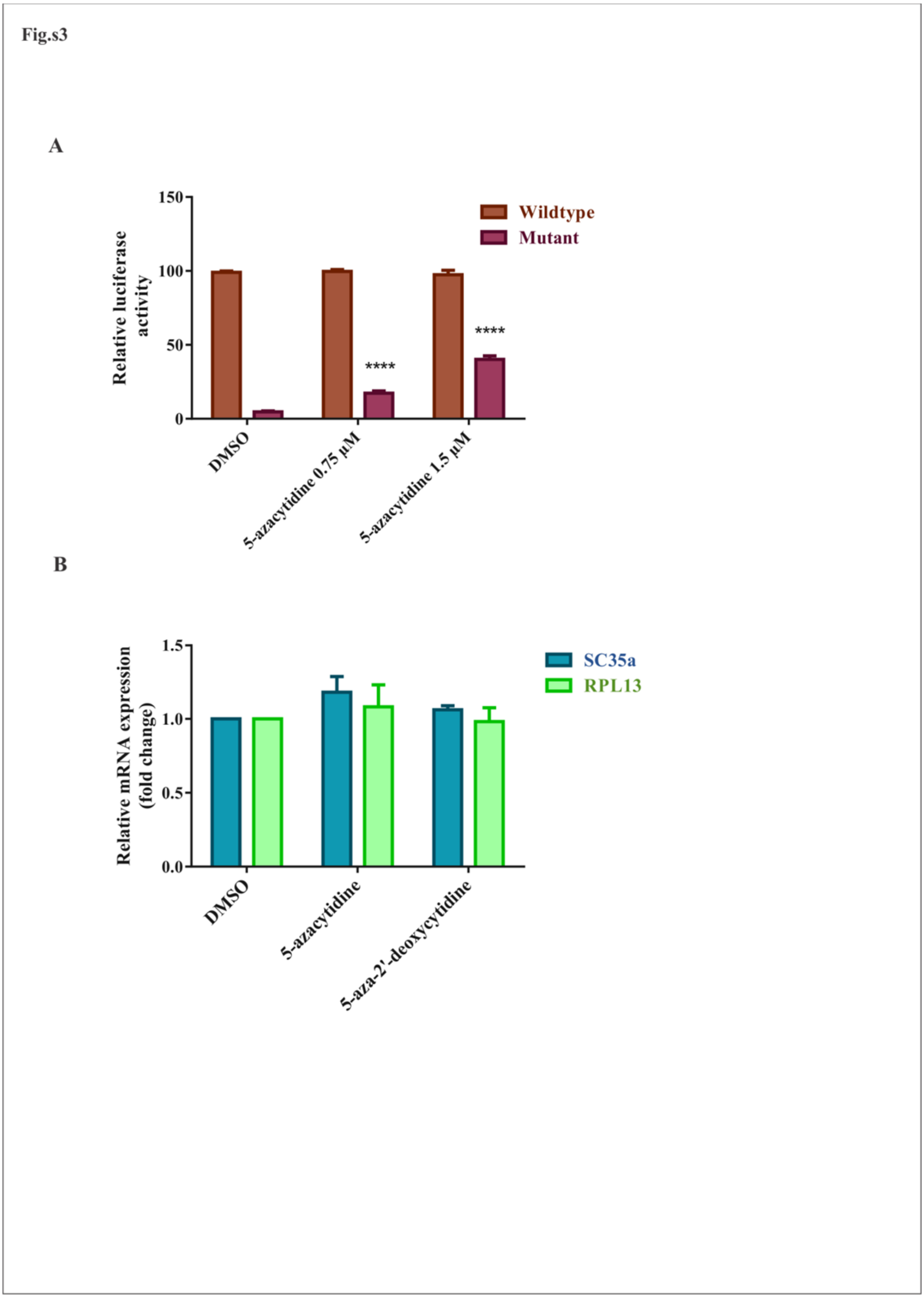

Supplementary Figure S3: 5-azacytidine does not affect the expression of NMD-insensitive isoforms of endogenous NMD targets.

(**A**) Percentage upregulation of NMD reporter luciferase activity in comparision with the wildtype following treatment with DMSO or 5-azacytidine (0.75μM or 1.56 μM) for 18 hours. Two-way ANOVA followed by Newman–Keuls multiple comparison test was performed to analyse the significance. N=3 and ****P<0.0001 for 5-azacytidine (0.75μM or 1.56 μM) treatment. Each bar represents average ± SD

(B) qRT-PCR analysis of the two NMD-insensitive RPL13 and SC35a mRNAs following treatment of HeLa cells for 18 hours with DMSO, 1.56 μM of 5-azacytidine or 1.56 μM of 5-aza-2'-deoxycytidine. The fold change on the y-axis represents the relative quantification of transcripts vs GAPDH mRNA, which is used as a normalization control. The signal detected in DMSO treated cells is set as 1. N=3 and Each bar represents average ± SD
