# Supplementary material for: 5-azacytidine inhibits nonsense-mediated decay in a MYC-dependent fashion
Source: EMBO Mol Med. 2014 Oct 15;6(12):1593–609. doi: 10.15252/emmm.201404461 (PMC4287977; doi:10.15252/emmm.201404461)

Figure. 1B

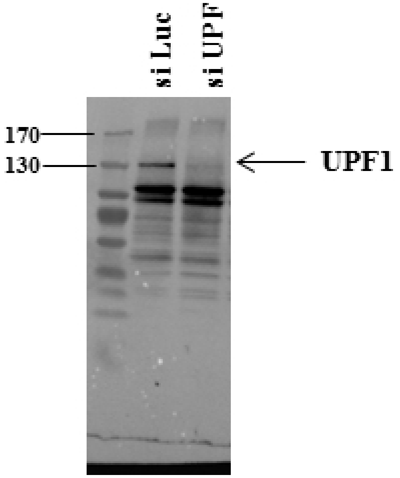

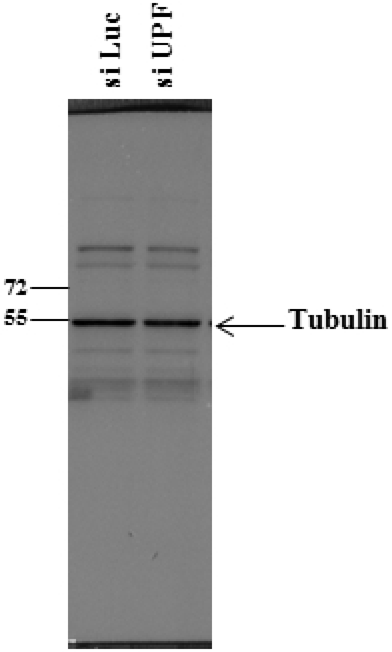

Supplement: Supplementary file 2 — Review Process File [file emmm0006-1593-sd2.pdf]
